# Of rAAV and Men: From Genetic Neuromuscular Disorder Efficacy and Toxicity Preclinical Studies to Clinical Trials and Back

**DOI:** 10.3390/jpm10040258

**Published:** 2020-11-28

**Authors:** Laurine Buscara, David-Alexandre Gross, Nathalie Daniele

**Affiliations:** 1 Genethon, 91000 Evry, France; lbuscara@genethon.fr (L.B.); dagross@genethon.fr (D.-A.G.); 2 Université Paris-Saclay, Univ Evry, Inserm, Genethon, Integrare Research Unit UMR_S951, 91000 Evry, France

**Keywords:** AAV, genetic neuromuscular disorders, gene therapy, clinical trials, toxicity, SMA, DMD, XLMTM

## Abstract

Neuromuscular disorders are a large group of rare pathologies characterised by skeletal muscle atrophy and weakness, with the common involvement of respiratory and/or cardiac muscles. These diseases lead to life-long motor deficiencies and specific organ failures, and are, in their worst-case scenarios, life threatening. Amongst other causes, they can be genetically inherited through mutations in more than 500 different genes. In the last 20 years, specific pharmacological treatments have been approved for human usage. However, these “à-la-carte” therapies cover only a very small portion of the clinical needs and are often partially efficient in alleviating the symptoms of the disease, even less so in curing it. Recombinant adeno-associated virus vector-mediated gene transfer is a more general strategy that could be adapted for a large majority of these diseases and has proved very efficient in rescuing the symptoms in many neuropathological animal models. On this solid ground, several clinical trials are currently being conducted with the whole-body delivery of the therapeutic vectors. This review recapitulates the state-of-the-art tools for neuron and muscle-targeted gene therapy, and summarises the main findings of the spinal muscular atrophy (SMA), Duchenne muscular dystrophy (DMD) and X-linked myotubular myopathy (XLMTM) trials. Despite promising efficacy results, serious adverse events of various severities were observed in these trials. Possible leads for second-generation products are also discussed.

## 1. Introduction

Neuromuscular disorders are a group of heterogeneous rare diseases characterised by skeletal muscle dysfunction and caused primarily by motoneuron, peripheral nerve, motor end plate or muscle deficiencies. This family of pathologies encompasses a wide clinical spectrum, ranging from very weak and barely detectable clinical signs to extremely severe and life-shortening forms. Common symptoms include muscle-specific patterns of atrophy and weakness, occasionally associated with the involvement of additional organs, the most common complication being cardiac and/or respiratory failure. These diseases can be caused by many factors, notably autoimmunity; inflammation; poisoning; toxin accumulation; tumours; environmental agents; neurologic, metabolic or traumatic syndromes [1,2,3]; aging [1,4]; and genetic inheritance or spontaneous mutations in muscle or nerve-essential genes. The large majority of mutations are monogenic, with every nature of mutation and transmission mode possible. The classification of neuromuscular diseases based on their origins and phenotypical features is published every year at http://www.musclegenetable. The 2020 update reports 1042 neuromuscular disorders caused by mutations in 587 different genes, classified in 16 groups, and many remain to be discovered [5]. The pathogenic mechanisms are very diverse, as they depend on the gene involved, and proteins with very different functions and subcellular localisations are affected (enzymes, structural proteins, metabolic key-players, etc). In this review, we will focus our interest on genetic neuromuscular diseases currently under interventional clinical trials with whole-body delivery.

## 2. Marketed Pharmacological Treatments

Before the 1990s, treatment options were limited to supportive therapies aiming at improving life comfort and lengthening lifespan. Anti-inflammatory drugs proved very efficient in preventing muscle degeneration and mortality in inflammatory myopathies [6]. Corticosteroids are also commonly used and show limited success in Duchenne muscular dystrophy (DMD, OMIM 310200), a very severe and the most common form of degenerative muscular pathology. Long-term clinical trials showed that prednisolone/prednisone or deflazacort corticosteroids reduce chronic muscle inflammation, stabilise muscle function, prolong ambulation and improve respiratory function and patients’ survival [7,8,9]. However, several side effects are associated with the prolonged usage of these immuno-modulators, the most severe being a drastic inhibition of the immune system’s functionality, occasionally leading to life-threatening opportunistic infections.

More recently, specifically targeted treatments were developed and approved for human applications by the regulatory agencies. The USA Food and Drug Administration (FDA) and the European Medicines Agency (EMA) approved Myozyme^®^ (α-glucosidase, Sanofi-Genzyme, Cambridge, MA, USA) for long-term enzyme replacement therapy in Pompe patients, who suffer from a severe metabolic myopathy caused by mutations in the α-glucosidase-encoding gene (glycogen storage disease type 2 or Pompe Disease, OMIM 232300) [10,11,12]. The treatment proved particularly efficient in improving lifespan and muscle, respiratory and cardiac functions in classical infantile-onset Pompe disease (<1 year of age, with cardiomyopathy) [13,14], with more contrasted results in late-onset forms of the disease [15,16]. Occasional infusion-associated reactions and adverse events related to the treatment were reported. Nearly all were resolved with an interruption or reduction of the infusion rate or symptomatic treatment. In almost every case, repeated bi-monthly intravenous injections of the product led to the generation of α-glucosidase-specific antibodies [13,14,15,16], although seldom showed evidence of in vitro inhibitory activity. Some patients developed anaphylactic shock [15].

Exondys 51^®^ (Eteplirsen, Sarepta Therapeutics, Cambridge, USA), a drug targeting the DMD pathology, was granted accelerated approval by the FDA in 2016 on the grounds of phenotype stabilisation, making it the first FDA-approved drug for DMD [17]. Severe Duchenne myopathy is caused by a variety of mutations in the dystrophin-encoding *DMD* gene [18]. The large majority are out-of-frame mutations resulting in the total loss of dystrophin, while the expression of shorter forms of dystrophin caused by in-frame *DMD* mutations leads to the milder Becker phenotype [19]. This observation constitutes the proof of concept that expressing shorter forms of dystrophin could be a therapeutic option for ameliorating, if not curing, DMD symptoms. Eteplirsen is a 30-nucleotide-long phosphorodiamidate morpholino oligomer (PMO) designed to skip *DMD* exon 51 and restore a shorter but functional reading frame. The weekly intravenous injection of this drug restores partial dystrophin expression in skeletal fibres [20], prevents muscle loss of function [21,22,23] and protects pulmonary and cardiac functions [24,25]. This drug offers a very good safety profile, probably due to its uncharged chemical nature. Of note, Translarna^®^ (Ataluren, PTC Therapeutics, South Plainfield, NJ, USA), a read-through RNA interference molecule targeting non-sense mutations in Duchenne, showed a weak benefit in DMD ambulatory patients in clinical trials [26,27] and was granted conditional approval for ambulatory patients by the EMA in 2014, but was refused by the FDA. The treatment proved safe and delayed ambulation loss in longer-term studies compared with a historical cohort [28].

Spinraza^®^ (Nusinersen, Biogen, Cambridge, MA, USA) was the first curative drug for spinal muscular atrophy (SMA) in paediatric and adult patients to be approved by the FDA in 2016 and the following year by the EMA [29,30]. SMA, the most common motoneuron degenerative disease and the leading genetic cause of infant mortality, is due to hereditary bi-allelic mutations in the *SMN* gene [31]. Spinraza^®^ is an antisense oligonucleotide interfering with the splicing of an alternative form of the gene (*SMN2*) and leading to the production of a functional SMN protein. Repeated intrathecal injections result in an increase in SMN proteins and meaningful improvement in motor development and function with the associated survival of the patients [32,33]. Just recently, in August 2020, the FDA approved Evrysdi^®^ (Risdiplam, Genentech/Roche, San Francisco, CA, USA) as the first oral and at-home treatment for all SMA patients from 2 months of age [34]. Similarly to Spinraza, this *SMN2* splicing modifier increases the levels of SMN proteins and shows clinically meaningful improvements in survival and motor and respiratory functions in SMA patients [35,36,37]. However, while Spinraza requires four administrations in the spinal cord a year, Evrysdi is taken orally for systemic distribution once a day, widening the field of application to patients excluded from intrathecal injections because of scoliosis.

Even though these drugs ameliorate the patient’s life and prognosis, they do not cure the diseases and necessitate constant re-dosing, a burdensome shortcoming for patients with an already altered quality of life. Long-term adverse events due to constant drug re-administration are also an important issue, especially as an immune response towards the treatment often develops with time, impeding its efficacy. Moreover, these personalised medicine treatments are generally highly specific for the targeted disease and mutation. Because of their wider range of application, a very intense research field is focused on developing gene replacement approaches. These strategies, which take advantage of the natural capacity of viruses to infect specific human cells, consist of inserting therapeutic genes in place of viral sequences in vectors devoid of replicative capacity. They offer the advantage of being usable regardless of the mutation type and position, at least for pathologies caused by losses of function. After a long period of difficulties linked mostly to the route of administration and to the production of the therapeutic vectors, the last ten years finally saw the translation of several proofs of concept into promising clinical trials. The vector favoured for the delivery of genes in neuromuscular tissues is derived from the adeno-associated virus (AAV). In the last ten years, several AAV-based treatments have been approved for human usage. In 2012, Glybera^®^ (alipogene tiparvovec, UniQure, Lexington, KY, USA) was the first to be accepted by the EMA for the correction of a rare inherited metabolic disorder, substantiating AAV innocuousness and long-term efficacy [38,39]. Luxturna^®^ (Voretigene Neparvovec, Spark Therapeutics, Philadelphia, PA, USA) was later approved for the local treatment of a rare retinal disease [40,41,42]. Very recently, regulatory agencies granted full (FDA) and conditional (EMA) approval to Zolgensma^®^ (onasemnogene abeparvovec-xioi, AveXis/Novartis, Bannockburn, IL, USA), the first AAV-based treatment for the whole-body correction of SMA [43,44,45], paving the way for other myopathies.

## 3. The Therapeutic Toolbox for Muscle Gene Therapy

### 3.1. About Wild-Type AAV

The AAV virus is a 25 nm-diameter non-enveloped human parvovirus, with a simple architecture composed of a single-stranded 4.7 kb linear DNA genome encapsidated within an icosahedral protein capsid. The DNA bears four open-reading frames (ORFs) coding, respectively, for the four non-structural Rep proteins involved in the viral cell cycle (Rep 78, 68, 52 and 40); the three structural Cap proteins VP1, VP2 and VP3, assembling in a 1:1:10 ratio to constitute the 60 monomers of the capsid; the assembly-activating protein (AAP), promoting capsid assembly [46]; and the recently described membrane-associated accessory protein (MAAP) [47]. The ORFs are framed by two highly structured 145 bp palindromic inverted terminal repeats (ITRs) acting in cis as structural signals to drive AAV replication and genome packaging. AAV can infect both dividing and quiescent cells [48].

Various AAV serotypes of human and primate origin (AAV1 to AAV13) and more than a hundred natural variants have been identified (AAV1 [49], AAV2 and AAV3 [50], AAV4 [51], AAV5 [52], AAV6 [53], AAV7 and AAV8 [54], AAV9 [55], AAV10 and AAV11 [56], AAV12 [57] and AAV13 [58]). Based on VP1-capsid composition, the AAVs were phylogenetically classified into six clades, regrouped together according to genetic relatedness [59]. Although many display a broad tissue tropism, they generally show preferential infections of specific organs. The cell tropisms depend on many parameters, but subtle differences in the capsid’s amino acid sequence and structure are one essential feature driving tissue targeting [55,60,61]. Once disseminated in the blood stream, AAVs have to overcome several barriers to deliver their DNA within host cells’ nuclei. First, AAVs can be neutralised by pre-existing neutralising antibodies (NAbs), as seroprevalence resulting from natural infections with wild-type AAV is common in the general human population, with a high cross-reactivity between serotypes [62,63,64]. Second, AAVs have to attach to specific receptors before being internalised within host cells. AAV capsids were shown to interact with specific glycan moieties of host membrane proteoglycans: heparan sulfate for AAV2 [65]; heparin for AAV3 and AAV6 [66]; sialic acid for AAV1, AAV4, AAV5 and AAV6 [66,67,68,69]; and galactose for AAV9 [70,71,72]. Transmembrane receptors such as PDGFR for AAV5 [73] and the 37/67 kDa laminin receptor LamR for AAV8 [74] were also reported to be surface receptors. Their in vivo biodistribution correlates with and could account for virus tropism. Efficient virus endocytosis requires secondary binding events with membrane co-receptors. For AAV2, the most widely studied AAV, the hepatocyte growth factor receptor c-Met [75], αVβ5 integrin [76] and fibroblast growth factor receptor 1 (FGFR1) [77] were demonstrated to increase AAV infectiosity and proposed as co-receptors. However, using a candidate approach based on genetic deletion and supplementation, Pilay et al. demonstrated the existence of a co-receptor common to all the tested serotypes (AAV1, 2, 3B, 5, 6, 8 and 9), the previously uncharacterised type I transmembrane protein KIAA0319L, renamed AAVR [78,79]. Within the cell cytoplasm, AAV undergoes intracellular trafficking via the microtubule network to reach the nucleus [80] and achieves endosomal escape, nuclear entry and capsid unfolding. The AAV lytic cycle needs co-infection with a helper virus such as adenovirus [49], herpesvirus [81] or cytomegalovirus [82] for replication to occur. In the absence of this helper virus, the AAV enters a latent state. Several reports have evidenced the preferential integration of the AAV genome into the transcriptionally active environment of the AAVS1 locus in the q13.4-Ter region of host chromosome 19 genomic DNA [83,84,85,86,87]. Other hotspots were evidenced in chromosome 5p13.3 (AAVS2) and chromosome 3p24.3 (AAVS3) [88]. However, these studies were performed in cell culture, and it was recently evidenced in vivo that AAV mainly persists as transcriptionally active episomal forms and sometimes integrates randomly in the host genome [89]. Clonal integration in six oncogenes in liver tissue associated with hepatic tumorigenesis was also identified [89,90]. No specific enrichment was found in major AAV targets previously identified in cell lines [89,90].

### 3.2. Of the Usage of Recombinant AAV for Central Nervous System (CNS) and Muscle-Specific Targeting

For neuromuscular diseases, AAV vectors stand out as the most promising tools for driving body-wide muscle gene expression, as their wild-type counterparts have not been associated with a pathologic condition, they target myocytes and they are relatively poorly immunogenic. Nonetheless, several factors limit their application, mainly their low packaging capacity (<5 kb), especially as many neuromuscular genes are larger. Another issue is the targeting specificity, as specific gene delivery is desirable to reduce the risk of toxic off-target effects.

AAV recombinant vectors (rAAVs) derived from wild-type viruses are devoid of viral genetic elements, apart from the two ITRs in between which the transgene of interest is inserted. Plasmids encoding Rep, Cap and a helper are brought in trans within an appropriate production cell line to achieve DNA packaging [91]. AAV2-based recombinant genomes have been packaged in many different capsid types, resulting in a wide collection of “pseudotyped vectors” (rAAV2/X, where X stands for the capsid serotype). In the absence of the Rep gene, in both murine models and cell lines, the rAAV genome mostly concatamerises and forms circular, transcriptionally active episomes unable to divide when host cells cycle [92,93,94], or integrates at a very low rate in the host genome, randomly [48,95,96,97] or in preferential regions: near chromosomal instability points or in CpG islands, active genes and regulatory sequences [98,99,100,101]. In cell lines, chromosomal rearrangements were observed near the AAV-host genome breaking points [102,103]. Importantly, in murine hepatocytes, rAAVs were also reported to integrate at a low frequency into chromosome 12, at the *Rian* locus (RNA imprinted and accumulated in the nucleus), upregulating neighbouring non-coding RNAs and genes [104,105]. This integration, suggested to participate in murine hepatocellular carcinogenesis, seems specific to neonate animals and to some genetic backgrounds, and was not seen in adult mice [106].

More than 20 years ago, rAAV2s were the first vectors to prove their efficacy for the efficient and persistent transduction of post-mitotic neuromuscular cells [95,107]. The local brain delivery of a reporter transgene placed under the control of a ubiquitous strong promoter resulted in neuron and, to a lower level, glial cell transduction in rodents [107]. The long-term transduction of muscle fibres was observed after the intramuscular injection of a reporter gene in wild-type mice and rhesus monkeys, pointing out the inter-species tropism of this vector [95]. However, rAAV2s have a preference for slow-twitch muscle fibres, which might restrict their therapeutic benefits [108]. Additionally, of all the serotypes identified to date, AAV2 is the most common target of pre-existing NAbs in human populations [62,63], which could potentially prevent effective transduction in most of the putative patients [109]. Finally, a side-by-side comparative study of rAAV1 to 9 carried out with a ubiquitously driven luciferase reporter transgene evidenced that rAAV2 is amongst the lowest for general and muscle-specific transduction after intravenous injection, the optimal administration route for myopathy [110]. Today, rAAV2 is mainly used for tissue-specific gene therapy, such as local brain injection in clinical trials aimed at CNS delivery for Parkinson’s disease [111].

Recombinant AAV1, 7, 8 and 9 showed higher muscle transduction than rAAV2 after local injection in mice [54,55,112,113,114] and dogs [115]. Muscle targeting was also achieved, though with lower efficacy with AAV5 [113] and AAV6 [114].

Apart from very few diseases in which a specific group of muscles are affected and can be targeted by local delivery, whole-body muscle transfer has to be achieved for myopathy treatments, and the delivery is usually performed by systemic administration, or specific cerebro-spinal fluid delivery in the case of CNS-specific pathology. The body-wide intravascular delivery of rAAV packaged with reporter genes confirmed the widespread dissemination and highest muscle tropism of rAAV1, 7, 8 and 9 in mice [54,110,116,117,118,119,120,121], dogs [115,122,123] and monkeys [119,124]. Conflicting publications report on rAAV9’s preferential tropism for fast fibres [120] or slow fibres [114], but as they were performed with different promotors and different murine genetic backgrounds, general conclusions cannot be drawn.

The vascular endothelium is a major barrier for rAAV tissue distribution. Its permeation through the use of vascular endothelium growth factor (VEGF) was once demonstrated to enhance tissue transduction with rAAV6, largely inefficient by the intravascular route [125], though this effect is lost at high doses of the vector, and ensuing attempts to use it failed [116]. Muscle ischemia, a feature associated with some myopathies [126], was also shown to improve muscle targeting, partly for the same reasons [127].

Skeletal muscles are composed of long-lived mature post-mitotic fibres and of satellite cells, a population of progenitors crucial for muscle regeneration. Ideally, for expression persistence, therapeutic vectors should target myofibres and satellite cells, but unfortunately, rAAVs are inefficient for satellite cell transduction [128]. However, even though the episomal DNA can be diluted by successive cell divisions during muscle growth or regeneration, the transgene genome was shown to be stable for years in terminally differentiated myocytes, leading to continuous transgene expression [95,124,129].

Heart targeting is essential for treating neuromuscular diseases with cardiomyopathic features. Interestingly, several reports showed that rAAV9s lead to the highest levels in heart muscle [110,118,119,130,131], although rAAV6’s superior cardiac efficacy was once reported [132]. Differences in the vector doses and systemic routes of administration are likely to account for this discrepancy. The cardiotropic properties of rAAV9s might originate, at least partly, from their specific binding to galactose receptors [70,71]. Interestingly, the intravascular delivery of rAAV9 is also an appealing strategy for CNS targeting, as this serotype is the most efficient for crossing the blood–brain barrier. Indeed, motoneurons and gIial cells were transduced in the spinal cords and brains of mice, cats and non-human primates [118,133,134,135,136,137,138]. This strategy is a safer alternative to local CNS delivery. This unique feature of rAAV9s could come from increased vascular permeability and/or from their attachment to specific receptors distinct from those of other serotypes, such as the galactose receptor [70,71,72]. Indeed, the crystallographic structure of AAV9 revealed the specificity of the capsid in regions associated with receptor attachment that could account for its unique cellular tropism [61].

Finally, serotypes AAV1–9 transduce the liver with very high efficacy in mice, dogs and primates [110,116,117,118,119,120,122,129,135,136], but apart from the transient elevation of the aminotransferase enzyme related to the expression of the GFP transgene [135], no serious adverse events (SAEs) related to the capsid were reported during the biodistribution studies. Capsid-specific NAbs commonly developed with both local and systemic injection, though the levels varied with the dose and route of administration, but no major immunotoxicity was evidenced [95,110,112,115,135,136].

### 3.3. Restricting Expression by Muscle and CNS-Specific Promoters

Apart from capsid choice, a careful selection of the transgene regulatory elements, especially the promoter, is essential for specific expression. Viral promoters, such as the cytomegalovirus (CMV) or the Rous sarcoma virus (RSV), have generally been used for proofs of concept in early muscular gene therapy development, as they allow broad and powerful transgene expression [139,140,141,142]. The CMV promoter is currently used in several clinical trials for Duchenne and Becker muscular dystrophies, sporadic inclusion body myositis and Pompe disease (NCT02354781, NCT01519349, NCT00428935 and NCT00976352) [143,144,145,146,147]. However, it is now known that eukaryotic cells progressively silence transgene expression driven by viral promoters as a result of an immune mechanism to shut off viral expression, limiting their use for gene therapy applications where the long-lasting expression of the transgene is crucial [148,149,150,151]. An alternative to limit transgene silencing is the use of eukaryotic constitutive promoters, such as the elongation factor 1α (EF-1α), phosphoglycerate kinase (PGK), ubiquitin C (UBC) or hybrid promoters such as the chicken β-actin promoter coupled with the CMV early enhancer (CAG promoter), which shows high levels of transgene expression [152]. Interestingly, the CAG promoter is currently being used in a gene therapy clinical trial aiming at treating SMA type 1 patients: it shows success in driving appropriate expression levels in target tissues, as clinically meaningful benefits are achieved [153,154,155,156]. Nevertheless, constitutive transgene expression, notably in antigen presenting cells (APCs), was reported to induce an immune response [157,158,159].

In order to minimise ectopic transgene expression, muscle-specific promoters such as muscle creatine kinase (MCK) [160], desmin (Des) [161] or α-myosin heavy chain (α-MHC) [162] have been developed, showing higher muscle specificity compared to constitutive promoters [161]. Transgene expression efficacy driven by the Des promoter was successfully demonstrated in 2014 in preclinical studies performed in murine and canine models of X-linked myotubular myopathy (XLMTM) [163] and has recently shown promising results in a clinical trial with meaningful improvements in neuromuscular and respiratory functions (NCT03199469) [164].

Despite specific gene expression, muscle-specific promoters usually do not allow a high level of transgene expression in muscle cells and have a large size, limiting the packaging capacity for the transgene. Therefore, different laboratories have developed truncated muscle-specific promoters by selecting specific regulatory sequences to optimise both promoter strength and muscle-specific expression [165,166,167,168]. In 2008, Wang et al. designed compact muscle-specific promoters by combining an 87 bp proximal basal MCK promoter with a double (dMCK) or a triple (tMCK) tandem of the modified MCK enhancer [169], leading to highly efficient shorter promoters of 509 bp and 720 bp, respectively [170]. These two hybrid promoters demonstrate high transgene expression in skeletal muscles (except for the diaphragm), with no expression in the brain or liver. Interestingly, the dMCK and tMCK promoters are not active in the heart, which could be an advantage for the gene therapy of muscular diseases without cardiomyopathies. Following a successful proof of principle of gene transfer efficacy using tMCK in mouse models of Charcot-Marie-Tooth neuropathy type 1A [171] and limb girdle muscular dystrophy (LGMD) type 2D [172], this promoter has moved forward to clinical trials for these diseases (NCT03520751, NCT01976091 and NCT00494195) [173,174]. However, both dMCK and tMCK were reported to show fast-twitch myofibre preferences, which could limit treatment efficacy depending on the pathology [170]. Inversely, the MHCK7 promoter (770 bp), based on the assembly of specific enhancer and promoter regions of MCK and α-MHC, was shown to direct high levels of transgene expression specifically in the skeletal and cardiac muscles, with the advantage of being expressed in both fibre types [175], and proved more efficient for muscle expression than MCK1 in a murine model of Pompe disease [176]. This promoter was shown to direct robust micro-dystrophin expression in a systemic gene replacement clinical trial for Duchenne muscular dystrophy [177]. Compact muscle-specific promoters were also designed by assembling multiple copies of myogenic regulatory elements of natural muscle promoters and enhancers. The synthetic muscle-specific C5.12 promoter was reported to present a 6- to 8-fold expression increase over the CMV promoter [178].

The use of tissue-restricted promoters has also revealed their ability to evade undesirable adaptive immune responses directed against the transgene product. A possible explanation for these results is the inhibition of transgene expression in transduced professional APCs. Cordier et al. previously showed that inserting the muscle-specific C5.12 promoter instead of the ubiquitous CMV promoter enables human γ-sarcoglycan expression in mice, probably impairing the anti-transgene immune response [179]. The same observation was made with α-sarcoglycan driven by the C5.12 promoter [180] or α-galactosidase A driven by the DC190 liver promoter for treating Fabry disease [181]. Another hybrid promoter, also based on the MCK enhancer and coupled with the SV40 promoter (MCK/SV40), resulted in the long-term sustainability of the transgene expression with a minimal cellular and humoral immune response compared to the ubiquitous CMV and CAG promoters, suggesting benefits for gene therapy applications with immunogenic transgenes [168].

Liver targeting is important to promote tolerance to the transgene product in order to lead to stable muscle expression [182,183]. In this context, Colella et al. designed a new tandem promoter enabling the expression of the transgene in the targeted muscle cells for treatment efficacy, as well as in hepatocytes to trigger immune tolerance to the transgenic protein [184]. To combine muscle-specific and hepatic transgene expression, both the apolipoprotein E enhancer (ApoE) and the human alpha-1 anti-trypsin promoter elements, known to allow tolerogenic transgene expression in the liver, were multiplexed with the muscle-specific C5.12 promoter. This approximately 1 kb hybrid promoter efficiently promotes transgene expression in muscles and prevents transgene immunity.

## 4. Translating Preclinical Studies into Clinical Trials

The achievable skeletal muscle, heart and CNS-specific targeting, together with the apparent safety of capsids, paved the way for the preclinical assessment of AAV-driven therapies for myopathies. Dozens of proofs of concept were made, but we will focus this discussion on the strategies that were translated into clinics for the whole-body treatment of SMA, DMD and XLMTM congenital myopathy. Nonetheless, Table 1 provides a general overview of the main clinical features and SAEs observed in all body-wide and CNS-targeted AAV-driven interventional clinical trials ongoing for neuromuscular disorders.

### 4.1. SMA Trial

The ubiquitous SMN protein plays a key role in RNA regulation, and its deficiency in SMA is associated with cell-specific pre-mRNA splicing defects, possibly accounting for the tissue selectivity of the pathology [191]. Indeed, lower motoneurons are the cells primarily affected by degeneration in SMA, but other tissues, in particular, the heart, are also occasionally affected [192]. Local CNS delivery performed by the intrathecal administration of an rAAV9-h*SMN* vector proved efficient in correcting the motoneurons pathology in mice at doses in the 10^13^ vg/kg range [193]. Widespread distribution of the transgene was also demonstrated in non-human primate (NHP) spinal cord and brain motoneurons. However, local delivery might be clinically risky, and limits body-wide vector distribution and the correction of extra-CNS symptoms. Interestingly, taking advantage of the ability of AAV9 to cross the blood-brain barrier after systemic administration, three independent laboratories reported the preclinical safety and efficacy of an rAAV9-human *SMN* vector in different animal models [194,195,196,197]. All these studies used a self-complementary (sc) vector, which bears a DNA construct enabling the shunting of the transcription of the second DNA strand and hence leads to quicker gene expression than conventional single-stranded vectors [198]. A remarkable rescue of the phenotype was observed in mouse and cat models of SMA receiving intravenous doses ranging from 3 × 10^13^ to 3.3 × 10^14^ vg/kg of body weight (see Table 2 for details). The treatments rescued survival and all the major clinical manifestations, such as muscle atrophy and weakness, respiratory distress, weight loss and paralysis. The correction of murine cardiomyopathy was also reported [195]. An extensive motoneuron distribution was confirmed in cynomolgus macaques [194]. The product used by Barkats and collaborators has similar effects to the one used by Kaspar and collaborators at a 10-fold lower AAV dosage [194,196] (Table 2). This might be due to the codon-optimised enhancement of the transgene expression and/or by way of promoter regulation. Widespread motoneuron transduction was observed in the spinal cord, and the heart, skeletal muscles and liver were also highly transduced. Apart from the necrosis of the tails and ears seen in long-term survivors and attributed to the lack of SMN in these tissues [194,196,197], no safety issues were evidenced, and a clinical trial was initiated in 2014 [44]. Fifteen 0.9- to 7.9-month-old patients were treated intravenously with an rAAV9-h*SMN* product controlled by the hybrid CMV enhancer/chicken-β actin promoter (product referred to as AVXS-101), either with 6.7 × 10^13^ vg/kg (three patients) or 2 × 10^14^ vg/kg (twelve patients) (AveXis/Novartis, NCT02122952) [44]. To this day, all the patients are alive and show significant amelioration of motor, respiratory and nutritional functions [44,153]. The improvements are substantial as seen from a comparison with a natural history cohort [154]. The effect is dose-dependent and related to the time of initiation: the earlier the injection, the more efficient the treatment [156]. This treatment proved more efficient than Spinraza^®^ [155]. It is also longer-lived and safer, as Spinraza^®^ necessitates constant re-administration by the risky intrathecal route [199]. Additional benefits might come from the widespread correction of SMN-related defects in other organs, the most important being the cardiac tissue. To date, fifty-six SAEs have been reported, amongst which two are deemed treatment-related [44]. They are limited to elevated serum aminotransferase levels reaching more than 10 times the normal range, without any other liver enzyme abnormalities or clinical manifestations. This important elevation of hepatic enzymes was rescued by a short course of glucocorticoids in the first patient, treated with a low dose (prednisolone, 1 mg/kg/day for 30 days, starting one day before AAV injection), which was thereafter administered systematically one day before the treatment administration to prevent liver-related toxicity. Granted these excellent results, the FDA approved AVXS-101 for usage in SMA patients in the USA in May 2019 [43]. This new drug goes by the name of Zolgensma and is the third AAV-based gene therapy approved to date for genetic diseases.

Intriguingly, a preclinical report published after this trial’s initiation demonstrates the acute toxicity of a closely related product composed of an identical CAG-h*SMN* cassette packaged in the rAAV9 variant AAVhu68 and injected into wild-type NHPs and piglets at 2 × 10^14^ vg/kg (the highest dose in the SMA trial) [200]. The biological abnormalities did not resolve in one out of three injected NHPs, leading to euthanasia at Day 5. No piglets died. The vector genome copy number was roughly 1000-fold higher in the liver than in other tissues. Intense dorsal root sensory neuron degeneration was evidenced in both the NHPs and piglets, with additional acute hepatocellular injury and liver failure, systemic inflammation and internal haemorrhage in monkeys. Because of the acute time course (abnormal parameters at Day 4–5), the toxic effects are not thought to be related to the activation of an adaptive immune response to the capsid or transgene and destruction of hepatocytes. They are more likely to result from the activation of an intracellular cellular stress pathway linked to genome or capsid overload in hepatocytes, together with the activation of systemic inflammation and the associated coagulopathy. In line with these findings, another AAV9-derived vector, AAV-PHP.P, coding for an unrelated GFP transgene and injected in NHPs at a slightly lower dose of 7.5 × 10^13^ vg/kg (nearly identical to the lower dose of 6.7 × 10^13^ vg/kg in the SMA trial) led to similar toxic effects on the liver and to thrombocytopenia and haemorrhage [201]. Here again, the time course of the acute symptoms is not consistent with an undesirable activation of the adaptive immune system. It is unclear whether liver damage or coagulopathy is the primary defect, but it is worth mentioning that the liver damage induced by some viral infections participates in lowering the platelet number, although this mechanism was not reported for AAV vectors [202]. Whether the toxic effects are related only to the dose or to the capsid used remains unclear. AAVhu68 and AAV PHP-B are closely related to AAV9 (two and seven amino acids of variation, respectively), but that could substantially change vector entry and processing. It would seem so, as high doses of rAAV9 ranging from 7.5 × 10^13^ vg/kg [201] to 1–3 × 10^14^ vg/kg [138] did not lead to toxicity in NHPs. The toxicity might also be related to the species used (NHPs and piglets) and the health status (wild-type animals) and will not necessarily translate into human toxicity in patients, as vector processing might be substantially different. The toxicity could also relate to the un-unified mode of vector purification and contaminants, especially as the ratio of empty/full capsids varies according to protocols. Thus, the therapeutic window is probably quite narrow in the SMA trial, as high levels of vector are necessary to achieve therapeutic benefit.

### 4.2. DMD Trials

DMD is a devastating and the most common muscle degenerative pathology, and as such, it has been the subject of many therapeutic attempts. Respiratory and cardiac complications are common, and patients’ lifespans are severely reduced. *DMD* is the largest human gene (≈14 kb cDNA, NM_004006.2), which impedes its encapsidation within an AAV. Dystrophin is composed of an N-terminal actin-binding domain, 24 spectrin-like repeats articulated by four hinge regions and, at the C-terminal extremity, a cysteine-rich domain and a specific C-terminal domain. This highly flexible molecule interacts with a membrane-bound molecular complex (DAPC for dystrophin-associated-protein complex) and with sarcomeric actin, ensuring the plasticity of the muscle structure and resistance to contraction-induced injury. The observation that the deletion of a large part of the central domain leads to a very mild phenotype in patients set the ground for a large number of therapeutic trials aiming at expressing dystrophin forms shortened in the spectrin-like region [19]. Two main paths have been followed: shortening the natural gene by inducing the skipping of specific exons and restoring the reading frame (mutation-specific therapies) or bringing in trans a reduced version of the gene (a therapy amenable to all forms of dystrophinopathies). A founder paper of Chamberlain’s team established the importance of the different domains by investigating the phenotypes of transgenic mice in which various forms of shortened dystrophin, named micro (<30% of the full-length coding sequence)- or mini-dystrophin, were expressed [203]. Exon-skipping feasibility and efficacy was demonstrated by bringing the adequate oligonucleotide within myofibres using a U7-driven AAV [204,205,206,207,208,209]. The gene transfer of micro-dystrophin offers more versatile options and proved very efficient in improving muscle force, protecting muscle from contraction-induced lesions and improving heart function in murine and canine models of the disease using the rAAV serotypes 6 [125,210,211,212], 8 [213,214,215,216] and 9 [139,141,217,218,219,220,221] delivered by the intravascular route. The most commonly used micro-dystrophins are variants of the ΔR4-R23ΔCT form, but the inclusion of the R16-R17 spectrin-like domains, involved in linkage to the membrane-bound cell metabolism regulator nitric oxide synthase NOS [222], was proposed to have additional therapeutic benefits [140,218]. Based on these preclinical proofs of concept, three clinical trials using micro-dystrophin gene transfer have been in progress since 2017 (Pfizer [New York, NY, USA], NCT03362502; Sarepta Therapeutics: NCT03375164; Solid Biosciences [Cambridge, MA, USA], NCT03368742). The three trials use muscle-specific promoters, rAAV9 or rAAVrh74 serotypes, and high and comparable doses of vector (1 × 10^14^ to 3 × 10^14^ vg/kg) (see Table 3). AAVrh74 was chosen by one group (Sarepta’s trial) because of its simian origin, which should decrease the likelihood of pre-existing immunity. Indeed, in a population of DMD patients, AAVrh74 sero-prevalence was shown to be low (measured in fewer than 20% of the patients tested) [223], and the average titres were also amongst the lowest [224]. Quite surprisingly, this seems to be a specific feature of DMD, as higher titres of antibodies are measured in non-DMD children, possibly owing to the small size of the population or to a disease-specific effect on AAV biology [224]. Another study even showed higher levels of antibodies against rAAVrh74 than against other serotypes in a healthy child population, probably because of cross-reactivity with serotypes present in humans [225]. Furthermore, AAVrh74’s safety has been demonstrated in a preclinical dose-escalation study in Duchenne’s model mice and in NHPs [226,227], as well as in humans in a clinical trial targeting LGMD, though the doses used were 100-fold lower than in the current DMD trial (1 × 10^12^ or 3 × 10^12^ vg/kg in the LGMD trial versus 2 × 10^14^ vg/kg in the DMD trial) [228]. The minimal effective dose was defined as 2 × 10^14^ vg/kg in *mdx* mice, a DMD model, and safety was confirmed in NHPs at doses reaching up to 6 × 10^14^ vg/kg [226,227,229]. One year after a single injection of 2 × 10^14^ vg/kg of AAVrh74-MHCK7-coΔR4-R23ΔCT (SRP-9001) in four patients, the first results are encouraging in terms of safety [177]. No SAEs were reported, and 18 mild or moderate events were deemed treatment-related. As previously observed in haemophilia [230,231] and SMA [44] clinical trials, liver enzymes peaked and diminished with a glucocorticoid course (*n* = 3). No adverse immune responses occurred, and, as expected, a transitory T cell response and the development of stable titres of antibodies against AAVrh74 were observed. The product was highly expressed, as seen in biceps brachii biopsies. Whether the treatment has any beneficial effects remains to be assessed more closely, although a clinically meaningful improvement of 2.2 to 7 points on the NorthStar Ambulatory Assessment score (NSAA) multi-parametric scale (maximum score of 34) suggests motor function improvement. A comparison with a historical cohort of untreated patients and longer time of treatments is needed to draw more definite conclusions. These encouraging results preclude dose escalation, and a new randomized, placebo-controlled clinical trial with a much larger sample size is under way (NCT03769116).

However, at very close doses, two other products composed of an rAAV9, a muscle-specific promoter and a micro-dystrophin transgene led to product-related SAEs. In one trial (product PF-06930026, Pfizer), the six participants included to date have shown a mean of ≈40% dystrophin-positive fibres at 1 × 10^14^ vg/kg and ≈70% at 3 × 10^14^ vk/kg in a bicep biopsy taken two months after injection, corresponding altogether to ≈24 to 30% of normal dystrophin expression [187]. The NSAA score increased by 4.5 points after one year in two participants treated with the lowest dose. However, one child treated with 3 × 10^14^ vg/kg developed a rapid antibody response with complement activation, acute kidney injury, haemolysis and thrombocytopenia. A transient 2-fold elevation of liver serum enzymes was observed, though it was not considered significant enough to indicate hepatic failure. Suspected complement-mediated nephropathy resulted in a protocol-driven pause of enrolment. Haemodialysis together with a course of complement inhibitor solved the problem in fifteen days. In the third trial (product SGT-001, Solid Biosciences), which differs slightly by the construct used (product SGT-001, different promoter and integration of the nNOS-binding domain in the transgene), similar treatment-related toxic events were seen in two patients at doses of 5 × 10^13^ and 2 × 10^14^ vg/kg. To date, six patients have been included, three at low and three at high doses. The preliminary results showed weak dystrophin expression in the three patients who received low dosages. The first patient injected at 5 × 10^13^ vg/kg developed complement activation, kidney failure and platelet count drops without signs of liver damage. The clinical hold [186] was lifted in 2018 after full symptom resorption following treatment with a modified course of steroids and a complement inhibitor and a change in the study design (the inclusion of an intravenous glucocorticoid administration in the first weeks following drug injection). A second patient dosed at 2 × 10^14^ vg/kg developed the same symptoms together with cardiopulmonary decline, leading to a second FDA hold of the trial. The SAEs fully resolved, but the clinical trial remained on hold on the grounds of remaining questions related to the mode of production of the product [185,232] and was finally allowed to continue in October 2020 [233]. A dose-finding study in a canine model of the pathology did not evidence any safety issue for this product at doses reaching 5 × 10^14^ vg/kg [221,234]. These SAEs could be related to the AAV9 capsid, though no severe side effects were observed in the SMA trial with this serotype at an equivalent dosage. The genetic background might account for the different effects between the SMA and DMD trials, whether for vector processing or the immune response. In the absence of liver injury, an immune response-mediated platelet drop, complement activation and ensuing nephropathy might be a reasonable hypothetical pathogenic mechanism. This could also be in line with the incidence of the age of the patients, as younger children are included in the only trial without SAEs, and the immune system is immature at a younger age [235]. The information on the three DMD trials is summarised in Table 3.

### 4.3. XLMTM Trial

XLMTM is a very rare congenital centronuclear myopathy caused by mutations in the *MTM1* gene, affecting 1/50,000 boys [236]. Skeletal and respiratory muscles are deeply affected, and many patients decease before one year of age, mainly from respiratory failure. The *MTM1* gene encodes a lipid phosphatase, myotubularin, involved in PI_3_P dephosphorylation and membrane remodelling [237,238]. The myotubularin cDNA, together with the regulatory elements, can be packaged in an AAV, and two very good murine and canine models of the disease recapitulate the main features of the pathology, noticeably, histological defects specific to centronuclear myopathies, generalised muscle hypotrophy and weakness, and lifespan reduction [239,240,241]. With these tools in hand, Buj-Bello and collaborators established a very convincing proof of concept, first by intramuscular injection with an rAAV2/1-CMV-*mtm1* product [242], and next using the whole-body delivery of an rAAV2/8-Des-*mtm1* product in mouse and canine models [163]. In both models, a single intravenous injection of a dose of ≈3 × 10^13^ vg/kg led to an important improvement of muscle and respiratory functions, and survival was largely extended. Importantly, therapeutic effects were also observed, though to a lesser extent, in older mice, showing that pathology reversal, essential in patients presenting the symptoms at birth, could be achievable. In 4-year-old, long-term survivor dogs, gait, respiratory and neurological functions remained comparable to the ones of wild-type, age-matched controls, despite a progressive decline in the vector copy number in muscles, which reached a plateau after three years of age, and a diminution of muscle force [243]. A dose study carried out in the canine model established the dose-dependency of the therapy, with a significant correction achieved from 2 × 10^14^ vg/kg, a quasi-normalisation of the phenotype at 5 × 10^14^ vg/kg and no significant side-effects, apart from the expectable humoral immune response towards the vector and a thickening of the heart septal wall without functional consequences [244]. In this protocol, muscle expression defects evidenced by a transcriptomics approach were corrected by the mid-dose of 2 × 10^14^ vg/kg [245]. Considering that the doses reversing the pathology are in the 10^14^ vg/kg range and challenge vector production, an additional efficacy study was carried out in three infant NHPs [246]. Eight weeks after intravenous injection, a dose of 8 × 10^14^ vg/kg did not lead to significant treatment-related adverse events and produced MTM1 protein expression at levels 8- to 20-fold higher than endogenous levels in target skeletal muscles [246]. Importantly, despite a high vector copy number in the liver, the myotubularin protein level remained normal, and serum markers of liver damage did not peak significantly. Altogether, these results led to the initiation of a clinical trial in 2017 on XLMTM infants. The ASPIRO phase 1/2 trial aims at treating ventilatory-assisted patients aged less than 5 years with ascending doses (1 × 10^14^ vg/kg or 3 × 10^14^ vg/kg) of an rAAV2/8-Des-h*MTM1* vector (AT132 product, Audentes Therapeutics [San Francisco, USA], NCT03199469). Until very recently, the results were strikingly positive. To date, twenty-three patients have been treated, six at 1 × 10^14^ vg/kg and 17 at 3 × 10^14^ vg/kg: the CHOP-INTEND (Children’s Hospital Of Philadelphia INfant Test of Neuromuscular Disorders) has improved by various levels, the limb and trunk strength have increased, and new developmental skills have been achieved, such as controlling head movement, rolling over or sitting unassisted [247,248,249]. Respiratory function has improved significantly resulting in patients being weaned off ventilators completely. One SAE possibly related to the product occurred and was resolved by a course of intravenous steroids and supportive care. However, since the 5 May 2020, three patients treated with the highest dose have died. All three patients had progressive liver dysfunction characterised by hyperbilirubinemia starting a few weeks after dosing. Preliminary findings suggest that two children died from sepsis and one from gastrointestinal bleeding. The FDA put the trial on hold on the 29 June [189]. This tragic event remains hard to rationalize, as 14 out of 17 children treated with the high dose have not developed complications to date. The common features of the three deceased children were an older age (the boys were at the higher end of the age cut-off), a heavier weight and a pre-existing hepatobiliary disease of an unknown severity, although one can assume it to be mild, as hepatic disorders were an exclusion criterion. This condition might have facilitated liver toxicity due to the large doses of vectors. This toxicity is reminiscent of the one observed in NHPs [200,201], and the activation of complement through the formation of vector–antibody complexes, which have been implicated in lethal systemic inflammation with an adenovirus vector [250], has been hypothesized [251]. Of note, some children dosed at 1 × 10^14^ vg/kg also had pre-existing liver disorders and did not develop the complications, despite being years out from treatment.

## 5. Improvement of the Therapeutic Toolbox

### 5.1. Towards Safer Next-Generation Muscle and CNS-Restricted AAVs

It is becoming increasingly evident that AAVs should be chosen carefully for every clinical application, considering specificities such as the patient’s genetic background, age, disease progression, sex, immunological state and targeted tissues. Capsid engineering is commonly used to develop safer next-generation AAV variants. These methods rely either on rational design in which capsids are tailored by targeted modifications, or on directed evolution, consisting of recovering new capsids from randomly generated high-complexity libraries after selective pressure on a tissue of interest. For neuromuscular disorders, the improvement of muscle transduction; reduction of off-targeting, especially in the liver; and development of vectors escaping the immune response are major endeavours.

AAV2.5, obtained by replacing five residues in the AAV2 capsid with corresponding orthogonal residues of AAV1 [146] and several other variants generated by variable combinations of 32 capsids’ amino acids [252], improved muscle transduction compared with parental serotype 2 or 1 but were not assessed for whole-body distribution. Three AAV2 variants, AAV2i8, a chimeric capsid obtained by replacing a receptor-binding hexapeptide motif in the AAV2 capsid with corresponding residues in the AAV8 capsid [253], and two variants obtained by peptide insertions in a hypervariable loop [254,255] showed equivalent or improved targeting in skeletal muscles, with an important reduction in the liver in comparison with AAV2. AAV2i8 was also shown to be less likely to be serum-neutralized than the parental capsid [253]. The ratio of skeletal muscle/liver transduction was also better than for AAV9 in mice [256] but not in NHPs [257]. An additional insertion of a galactose-binding footprint on AAV2i8 did not improve the ratio further in mice [256].

Three other variants proved even more efficient than AAV9 for improving the muscle/liver transduction ratio: (1) AAV-9.45 is an AAV9 variant obtained by the random integration of amino acids and showing reduced liver expression and identical muscle and heart transduction when compared with AAV9 [258]. (2) AAVpo1 is a natural pig isolate that transduces muscles and the heart to a slightly lower level than AAV9 but presents the advantage of being completely detargeted from the liver [259]. (3) AAV-B1 is a chimeric AAV isolated from a shuffled library consisting of 11 parental serotypes and displaying reduced liver transduction and at least 10-fold higher muscle and CNS tropism than AAV9 [260].

A series of mutations on surface phosphorylable residues of the AAV1 and AAV9 capsid improved vector stability and led to 3 to 10 times lower transduction in the liver than in muscles [261]. AAVM41 was isolated from a chimeric AAV1 and AAV9 capsid’s shuffled library and reduced both skeletal muscle and liver targeting while preserving heart transduction compared with AAV9, suggesting that this serotype could be of interest for rescuing cardiac pathologies [262,263]. Tyrosine-specific modifications of the AAV6 capsid can improve vector muscle entry [264].

Several variants demonstrated interesting characteristics regarding immune evasion. Bat AAV serotype 10HB transduced muscle with a higher muscle/liver ratio than primate AAV and showed a reduced sensitivity to antibody neutralisation [265]. A method consisting of purifying new AAV2 variants by rabbit antibody-specific affinity chromatography resulted in the identification of several antibody-resistant clones, though neither the relevance to human sera nor variant biodistribution were assessed [266]. The AAV1 variant CAM130 isolated through multiple rounds of neutralizing-antibody escape from several species evaded neutralizing antibodies, even at high concentrations, in mice, NHPs and human sera, while maintaining the tissue tropism of the parental AAV1, suggesting it could be suitable for clinical trials in large populations, as seropositivity is a common exclusion criterion [267]. Finally, after applying the double selection of variants resistant to human-serum neutralization and selected after local muscle transduction, the AAV mutant MuS12 was isolated and showed immune response escape together with the preservation of muscle tropism, although transduction was largely reduced by the intravenous route in comparison with AAV9 [268]. Considering that this variant transduces muscle differently according to the route of administration, low vascular permeability was hypothesised. Future protocol improvement could aim at selecting new variants after intravascular injection.

Altogether, these new vectors have the potency to improve targeting efficiency and reduce the off-target effects and immune response. Their respective characteristics are summarised in Table 4.

### 5.2. Enhancing the Repertoire of Muscle and CNS-Restricted Promoters

With the development of in silico analysis technologies, a multistep, genome-wide data-mining strategy was performed to identify conserved skeletal muscle-specific cis-regulatory modules (Sk-CRMs) in highly expressed muscle-specific genes. Sk-CRM4, containing binding sites for the E2A, CEBP, LRF, MyoD and SREBP transcription factors, boosted transgene expression driven by Des or C5.12 promoters in heart and skeletal muscles (up to 400-fold), with a significant improvement of the *mdx* mouse phenotype [269]. Similarly, a 1030 bp modular muscle hybrid (MH) promoter composed of two enhancers (from the *Des* and *Mck* genes, respectively), a proximal promoter and an intron (modified from the *Mck* gene and core promoter) was more efficient than the Des promoter in skeletal and cardiac muscles, with limited expression in non-muscle tissues compared with the CMV promoter, showing a high potential for muscular gene therapy [270].

The presence of large promoters limits the size available for the transgene in the cassette, which proves problematic for several muscle genes. Promoterless cassettes were recently tested for liver expression. In this strategy, the transgene, flanked by homology arms, is brought into the cells by rAAV and integrates by nuclease-free homologous recombination downstream of the native promoter, where it is regulated like the endogenous gene is. Despite promising results in hepatocytes [271,272], the promoterless strategy might be limited for muscle application, as muscle cells are mostly quiescent and homologous recombination is restricted. Nonetheless, it might prove interesting for satellite cell targeting, provided that muscle progenitor targeting can be achieved.

### 5.3. Detargeting with miRNA-Based Elements

While regulatory elements such as introns, polyA signals or the Woodchuck hepatitis virus post-transcriptional regulatory element (WPRE) can be added to improve global transgene expression, miRNA-based sequences can mitigate tissue-specific transgene expression [273]. MiRNAs are small (approximately 22-nucleotide-long) non-coding RNAs post-transcriptionally silencing gene expression in plants and animals. Once bound to complementary target sites (TS) in mRNA, they either reduce its stability or inhibit its translation, which results altogether in the reduction of protein expression [274]. While the number of identified miRNAs has constantly increased since their discovery in *Caenorhabditis elegans* in 1993 [275], the miRBase database reports 1917 annotated hairpin precursors and 2654 mature sequences in the human genome [276]. Some miRNAs present a tissue-specific pattern of expression, with expression detectable only in a particular tissue or at least 20-fold higher than in other tissues [277]. Amongst tissue-specific miRNAs are found the MyomiRs, a family of miRNAs expressed in both cardiac and skeletal muscles, namely, miR-1, miR-133a, miR-122a, miR-124a, miR-208b, miR-499 and miR-486, with the exception of miR-208a and miR-206, which are specifically expressed in the heart and skeletal muscles, respectively [278].

One strategy to improve the specificity of AAV-mediated gene delivery, overriding the broad tissue tropism of AAV vectors and/or promoter leakage in non-targeted tissues, is based on the miRNA-mediated post-transcriptional regulation of the transgene. Indeed, the insertion of miRNA TS into the 3′UTR of a gene expression cassette limits transgene expression in tissues expressing the corresponding miRNA [279]. Due to the small size of miRNAs, it is therefore feasible to insert different miRNA TS in the 3′UTR of the expression cassette to detarget specific cell types depending on the application. For neuromuscular disorders, this strategy was applied for the reduction of expression in the heart [280], liver [281,282] and APCs [283,284,285,286,287].

The control of heart transgene expression is of utmost importance, because even if no specific cardiac toxicity has been reported to date in clinical trials, preclinical reports have evidenced the danger of transgene cardiac overexpression when the heart is not the primary target [280] or even when it is [288]. The insertion of the cardiac-specific miR-208a TS in the cassette was shown to prevent cardiac transgene expression and rescued the cardiac toxicity resulting from transgene overexpression in this organ, while maintaining the efficient expression of the transgene in skeletal muscles [280].

MiR-122 is highly expressed in the liver. The insertion of miR-122 TS in the 3′UTR of a reporter gene was able to prevent protein expression in the liver after rAAV9 intravenous administration without interfering with cardiac protein expression [281,282]. The level of transgene repression was related to the number of repetitive miRNA TS used. However, the recent paper of Kraszewska et al. challenges this apparently safe approach. Indeed, in some genetic backgrounds, transgene expression was completely repressed not only in the liver, but also in the cardiac muscle, linked with the presence of miR-122 in these animals’ hearts. MiR-122 was also shown to be present in the human cardiac tissues of patients with cardiomyopathy and in human iPSC-derived cardiomyocytes. The cardiac expression showed high variability between different mouse strains, sexes and human individuals [289]. This publication challenges the liver-specificity of miR-122 and warns against miRNA inter-individual variability.

As previously mentioned, preventing transgene expression in APCs may avoid undesirable adaptive immune responses directed against the transgene product. A miR-based approach aiming at inhibiting transgene expression in APCs by inserting four targets of the endogenous miR-142-3p (exclusively expressed in the hematopoietic lineage) at the 3′ end of the transgene coding sequence [283] allowed escaping a deleterious adaptive immune response after gene delivery with either lentiviral [284] or AAV vectors [285,286,287].

Most importantly, it is crucial to verify during preclinical studies that miRNAs are not reduced by the miRNA TS and that their natural targets are not misregulated, as that could induce detrimental side effects. To our knowledge, no clinical trial has used miRNA TS in the cassette to date. If it ever happens, checking beforehand the mean level of the targeted miRNA in the treated population will be essential, as miRNA expression can substantially vary between individuals, sexes or pathologies [289,290,291].

## 6. From Preclinical Studies to Clinical Trials… and Back: General Point of View

**Figure 1:**
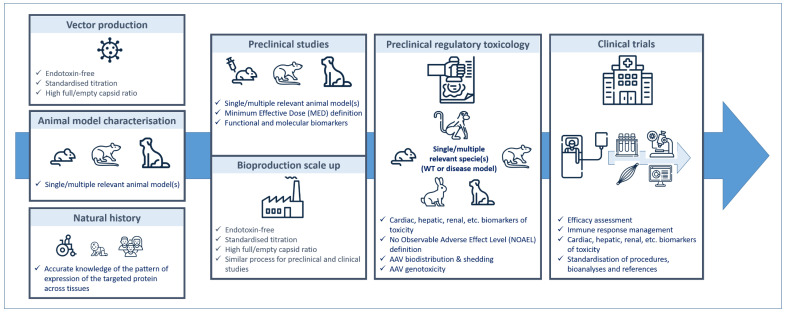
Of rAAV and men: the ideal journey.

The ongoing clinical trials summarised herein show spectacular results in terms of efficacy, especially in SMA and XLMTM, two very severe conditions characterised by generalised muscle weakness and respiratory deficiency often leading to infant deaths in the first years of life. The DMD trials need further investigation. However, this very beautiful landscape has lately been obscured by SAEs in two DMD and in the XLMLM trials, leading to three children’s deaths in the last case. It is hard to find common features in the two situations, as, apart from the doses, which are very close, the genetic background, the age of the patients and the vector capsids are different. DMD-related adverse events have been proposed to be caused by adverse immune reactions driving acute kidney failure, while XLMTM fatal hepatotoxicity is not associated with obvious immunotoxicity. Future investigations will undoubtedly document these side-effects and help with the design of next-generation products, but for now, with the current state of our knowledge, a lot of effort has to be put into designing the safest therapeutic strategies for future trials, especially as some diseases are not prone to being good candidates in terms of the benefit/risk ratio. Several factors have to be considered in the “ideal” trial design (see Figure 1).

### 6.1. Defining the Product

The transgene definition is obviously central and often evident, except in large proteins, where shorter forms have to be assessed with extreme care in preclinical studies to choose the best product possible. However, improvements can be made even with a full-length transgene. For example, codon optimisation leading to better protein expression has proven beneficial [196,292]. A thoughtful choice of promoter and other regulatory elements, such as WPRE addition, which proved efficient in enhancing transgene expression [293], can also improve the efficacy of the products. As for capsids, the choices are widening deeply, with new attractive vectors improving specific targeting. However, these vectors have to be assessed more closely in terms of safety before they can be considered for clinical usage. All these choices are crucial for specific tissue targeting and the reduction of off-target effects. Fundamentally, a very good knowledge of the levels of the protein to replace in every tissue of the healthy population is necessary to design the best targeted product. The addition of miRNA TS to detarget the liver [281], heart [280] and APCs [294] proved efficacious in reducing ectopic expression, but has not been tried in neuromuscular-deficient patients to date, probably because the underlying risks have to be assessed more closely. It is also worth mentioning that even with perfect targeting, the overexpression of transgenic protein in the targeted organs can also lead to toxic effects [295].

### 6.2. Manufacturing AAV

Vector production is a critical process for gene therapy success and safety. Importantly, the methods used for vector production and titration are not standardised, complicating the comparability of different clinical trials. Indeed, it was previously shown that the same production of rAAV8 led to significant variations in titres when dosed in 16 different laboratories [296]. In the absence of consensual methods, a common standard used for all clinic-intended rAAV production could help to correct the titres amongst trials.

Moreover, according to the method of production/purification used, various quantities of toxic contaminants can be found in AAV production. Indeed, endotoxins are known to be able to activate the human immune system and lead to SAEs and often contaminate AAV production. It is therefore crucial to reduce their load in the final product [297]. Their safety limit is defined as 5.0 International Units of endotoxin per kilogram of body mass by the FDA and the European Pharmacopeia for intravenous usage in humans [298,299], but could be raised further depending on the patient status (age, disease severity, etc). Of note, other contaminants, not known or tested, could also play a role in the safety and/or efficacy of the product. Notably, the presence of empty capsids in the final product was shown to reduce transduction efficiency and may participate in side effects [300].

### 6.3. Choosing the Best Preclinical Models

While proof-of-concept and preclinical studies aim at determining the minimal effective dose of the product, toxicology studies evaluate its safety/toxicity. Although using relevant animal models mimicking the human disease in proof-of-concept and preclinical studies seems obvious, the choice of animal models in toxicology studies remains unclear. The assessment of chemical drug toxicity is traditionally performed on wild-type animals such as rats, dogs or monkeys, as they are relevant for phase 1 clinical trials aiming at determining the safety in the general population. However, gene therapy cannot ethically be tested in healthy individuals. The crucial question is the relevance of extrapolating toxicology findings to human clinical trials. Vector entry relies on cell surface receptors and co-receptors, and its internal traffic requires components of the host cells. As these processes are not well deciphered in humans, inter-species variability could preclude toxicity results in some species. In line with this idea, piglets did not show liver failure and haemorrhage when administered the same dose of rAAVhu68 as NHPs [200]. Additionally, NHPs are not necessarily the best species as assumed, since toxicology studies in XLMTM at 8 × 10^14^ vg/kg did not detect coagulation defects or acute toxicity [246], while three children died at 3 × 10^14^ vg/kg. This is not completely surprising, as membrane and cytoskeletal remodelling likely alter vector processing in neuromuscular diseases, possibly significantly modifying AAV efficacy. In line with these considerations, the International Council for Harmonisation of Technical Requirements for Pharmaceuticals for Human Use (ICH) defines a relevant species as “*one in which the test material is pharmacologically active*” and states that if “*no relevant species exists, the use of relevant transgenic animals expressing the human receptor or the use of homologous proteins should be considered. […] In certain cases, studies performed in animal models of disease may be used as an acceptable alternative to toxicity studies in normal animals*” [299].

### 6.4. Defining the Dose

For intravenous administration, the AVV dose administered is proportional to body weight, regardless of the age, gender, genetic background and disease severity of the individual treated. However, any of these factors could influence AAV efficacy and toxicity. The relative weight of organs is not proportional to body weight during development. For example, the liver/body ratio is higher in children than in adults [301], which could lead to variable levels of transduction and influence vector biodistribution at different ages. The immune systems of young children are not fully mature [235] and could therefore facilitate AAV transfer. Although it is not the only difference, the two DMD trials showing toxicity events potentially linked to immune responses were performed in older patients than the trial without any SAEs. Sex was also shown to impact AAV transfer in hepatic tissue but not in other tissues, with the male liver being more transduced than females’ [302]. Finally, the disease itself can modify the structure and function of various organs, with high variability between individuals of the same age. Indeed, in the XLMTM trial, the three deceased patients had pre-existing hepatobiliary diseases and were older than the other infants without SAEs treated at the same dose. Although it is challenging, finding a better and universal normalisation method for dose calculation could probably improve clinical trial standardisation and safety.

### 6.5. Circumventing Immune Response

As previously mentioned, the adaptive immune response directed against a viral-derived vector restricts the full therapeutic potential of in vivo gene therapy [303]. Thus, in the first clinical trial showing safe and efficacious liver targeting with an rAAV2 vector carrying the human factor IX transgene (under the control of a liver-specific promoter), transgene expression was only transient [109]. A decline in expression starting at four weeks was associated with transient liver transaminases and the detection of CD8+ T cells directed against the AAV2 capsid. This unexpected deleterious cellular immune response was vector-dose-dependent and, in the absence of preclinical animal models, is still poorly understood. Nevertheless, a short prednisolone treatment quickly given in response to liver injury is often sufficient to stabilise transgene expression and has been used since then [230,231]. As a result, anti-AAV neutralizing antibodies are one of the most important remaining barriers, either impairing the efficacy of gene transfer in a set of patients with a cross-reactive pre-existing immunity against wild-type AAV, or precluding the redosing of patients developing a rapid and strong humoral response after the first vector injection. In preclinical models, numerous strategies targeting the host have been proposed such as plasmapheresis [304], direct tissue injection or isolated organ perfusion and immunosuppression combining rituximab (anti-CD20 depleting monoclonal antibody) and others drugs, or synthetic particles encapsulating rapamycin [305,306]. Other strategies target the rAAV vector itself, such as the use of alternative and less-prevalent serotypes, empty decoy capsids [307], exosome-enveloped AAV vectors or the generation of novel AAV capsids with optimised biodistributions and transduction efficacy, as well as the capacity to evade NAbs, as discussed above [47,305]. The most promising approach to including patients non-eligible to date was recently reported with the use of an IgG-cleaving endopeptidase from *Streptococcus pyogenes* (IdeS) [308]. The IdeS enzyme very rapidly (in a few hours) cleaves human IgG into F(ab′)2 and Fc fragments, and is safe and efficient in patients with donor-specific antibodies undergoing kidney transplantation [309]. In both mouse and NHPs, Leborgne et al. reported that IdeS treatment was able to decrease pre-existing anti-AAV antibodies to a level sufficient to enable efficient liver gene transfer, even in the setting of vector re-administration [308]. Equivalent properties were demonstrated with IdeZ, a homolog of IdeS [310]. In July 2020, Sarepta Therapeutics announced an agreement with Hansa Biopharma to develop and promote imlifidase (the commercial IdeS) as a pre-treatment for DMD and LGMD gene therapy.

### 6.6. Assessing Long-Term Efficacy

Since the clinical trials using whole-body delivery in neuromuscular diseases are quite recent, the long-term assessment of efficacy will be made available in the next few years. For CNS-targeted treatments, a relative stability of the treatment is to be expected, as neurons are the longest living cells of the body. However, muscles, while being in a post-mitotic state, are remodelled during growth and following exercise, which could dilute the therapeutic effect. Targeting the treatment to satellite stem cells could hence be useful to help maintain long-term efficacy. Unfortunately, AAV-driven attempts to target satellite cells have failed. While reinjection might prove complicated, it might be worth using lentiviral vectors to target stem cells, as it was demonstrated efficient in transducing satellite cells in vivo [311], or new rAAV-rDNA integrating vectors, which proved their efficacy for directed integration in dividing and quiescent cells [312]. Importantly, it was recently shown that the AAV virus is found in episomal and randomly integrated transcriptionally active forms in human samples of liver tissues, and while it is impossible to know the time of infection, considering the large number of samples, it certainly suggests long-term persistence of the virus [89]. Whether this is also true for rAAV genomes remains to be determined.

### 6.7. Assessing Long-Term Toxicity

Serious concerns about the long-term safety of rAAV vectors were raised after several genotoxic studies performed in mice. Recombinant AAV2, 8 and 9 vectors were shown to integrate in the *Rian* locus on chromosome 12, irrespective of viral transgene, mouse genotype, sex or genetic background [104,105]. This insertion upregulates non-coding RNA and genes proximal to the *Rian* locus and is associated with an increased rate of hepatocellular carcinoma (HCC). The trans-regulatory elements carried by the vectors influence genotoxicity: sometimes, insertion is seen, but adjacent oncogenes are not overexpressed and HCC does not develop. Capsid-specific properties may also influence genotoxicity. These results were all obtained in neonatal mice, and neither integration in the *Rian* locus nor HCC were ever observed in older animals [106]. Other results obtained with sc vectors evidenced insertion within proto-oncogenes injected in young adult mice [313]. As no liver tumours have been seen to date after rAAV treatment in humans, the risk of insertional mutagenesis is probably very low, if it ever exists. Nonetheless, patients should be followed longitudinally to monitor long-term effects. 

Figure 1 summarises the main steps necessary to push forward an AAV-based gene therapy medicinal product from preclinical studies to clinical trials

Without minimising the importance of the tragic toxic events seen in the current clinical trials, it is worth emphasizing that AAV-mediated gene therapy is the only treatment that led to highly significant disease improvement in severely affected human patients. It is now necessary to go back to bench work in order to decipher the pathogenic mechanisms underlying AAV-linked toxicity and design safer next-generation therapeutic cassettes. Indeed, AAV therapy remains the main source of hope for patients affected by neuromuscular disorders, and there are many more diseases to treat. Importantly, our laboratory is planning a new rAAV-based clinical trial using micro-dystrophin transfer in DMD patients, in partnership with Sarepta [314]. The baseline study, aiming at collecting data on the natural disease course in DMD male subjects aged from 5 to 9 years of age, is currently ongoing (GNT-014-MDYF, NCT03882827), and the interventional gene therapy trial should start in early 2021.

## Figures and Tables

**Table 1:** Summary of current body-wide and central nervous system (CNS)-targeted adeno-associated virus (AAV)-mediated gene replacement therapy clinical trials for neuromuscular diseases, as reported in *ClinicalTrials.gov*.

	PRODUCT/ADMINISTRATION					CLINICAL DESIGN				SERIOUS ADVERSE EVENTS
DISEASE	AAV Serotype	Promoter	Transgene	Name	Administration/Dose	Clinical Trial ID (Study Name) Sponsor/Collaborator	Study Phase/Status	Study Timelines (Clinical Follow-Up)	Age, Gender, Actual or Estimated/ Planned Number of Participants Enrolled	
DMD	AAV9	CK8	Micro-dystrophin	SGT-001	Intravenous 2 doses	NCT03368742 (IGNITE DMD) Solid Biosciences, LLC	Phase ½ active, not recruiting	2017–2024 (2 years)	4 to 17 years, males, *n* = 16/same as current	Complement activation kidney failure, platelet count drop (*n* = 1 at 5 × 10^13^ vg/kg) + cardiopulmonary insufficiency (*n* = 1 at 2 × 10^14^ vg/kg) [185,186]
DMD	AAVrh74	MHCK7	Micro-dystrophin	SRP-9001	Intravenous 2 × 10^14^ vg/kg	NCT03375164 Sarepta Therapeutics, Inc.	Phase ½ active, not recruiting	2018–2021 (3 years)	3 months to 7 years, males, *n* = 4/12	No serious adverse events [177]
DMD	AAVrh74	MHCK7	Micro-dystrophin	SRP-9001	Intravenous 1 dose	NCT03769116 Sarepta Therapeutics, Inc.	Phase 2 active, not recruiting	2018–2026 (5 years)	4 years to 7 years, males, *n* = 41/24	-
DMD	AAV9	Human muscle-specific	Mini-dystrophin	PF-06939926	Intravenous 1 × 10^14^ vg/kg 3 × 10^14^ vk/kg	NCT03362502 Pfizer	Phase 1B active, not recruiting	2018–2026 (5 years)	4 years and older, males, *n* = 30/12	Antibody response, complement activation, acute kidney injury, haemolysis, thrombocytopenia (*n* = 1 at 3 × 10^14^ vg/kg) [187]
SMA	AAV9	Hybrid CMV enhancer/chicken β-actin promoter	Human SMN	AVXS-101	Intravenous 6.7 × 10^13^ vg/kg 2 × 10^14^ vg/kg	NCT02122952 AveXis, Inc.	Phase 1 completed	2014–2017 (2 years)	Up to 6 months of age, males and female, *n* = 15/9	Elevated serum aminotransferase levels (˃10× normal level) [44]
SMA	AAV9	Hybrid CMV enhancer/chicken β-actin promoter	Human SMN	AVXS-101	Intravenous therapeutic dose	NCT03306277 (STR1VE) AveXis, Inc.	Phase 3 completed	2017–2019 (18 months of age)	Up to 6 months of age, males and females, *n* = 22/15	-
SMA	AAV9	Hybrid CMV enhancer/chicken β-actin promoter	Human SMN	AVXS-101	Intrathecal 6 × 10^13^ vg 1.2 × 10^14^ vg 2.4 × 10^14^ vg	NCT03381729 (STRONG) AveXis, Inc.	Phase 1 suspended (on clinical hold pending further discussions regarding pre-clinical findings)	2017–2021 (15 months)	6 to 60 months of age, males and females, *n* = 51/27	SAE mainly related to the disease itself (*n* = 7). Transaminitis events probably related to treatment (*n* = 2). [188]
SMA	AAV9	Hybrid CMV enhancer/chicken β-actin promoter	Human SMN	AVXS-101	Intravenous	NCT03461289 (STRIVE-EU) AveXis, Inc.	Phase 3 completed	2018–2020 (18 months of age)	Up to 6 months of age, males and females, *n* = 33/30	-
SMA	AAV9	Hybrid CMV enhancer/chicken β-actin promoter	Human SMN	AVXS-101	Intravenous 1.1 × 10^14^ vg/kg	NCT03505099 (SPR1NT) AveXis, Inc./PRA Health Sciences	Phase 3 active, not recruiting	2018–2021 (18 and 24 months of age)	Up to 42 days, males and females, *n* = 30/44	-
SMA	AAV9	Hybrid CMV enhancer/chicken β-actin promoter	Human SMN	AVXS-101	Intravenous single dose	NCT03837184 AveXis, Inc./PRA Health Sciences	Phase 3 active, not recruiting	2019–2021 (18 months of age)	Up to 6 months of age, males and females, *n* = 2/6	-
XLMTM	AAV8	Des	Human MTM1	AT132	Intravenous 1 × 10^14^ vg/kg 3 × 10^14^ vg/kg	NCT03199469 (ASPIRO) Audentes Therapeutics	Phase ½ active, not recruiting (FDA placed on clinical hold since June 2020)	2017–2024 (5 years)	Up to 5 years, males, *n* = 24/12	Progressive liver dysfunction, hyperbilirubinemia, death from sepsis or gastrointestinal bleeding (*n* = 3/17 at 3 × 10^14^ vg/kg) [189]
Pompe	AAV2/8	Liver-specific promoter	hGAA	ACTUS-101	Intravenous 2 doses	NCT03533673 Asklepios Biopharmaceuticals, INC./Duke University and National Institute of Arthritis and Musculoskelatal and Skin Diseases (NIAMS)	Phase ½ recruiting	2018–2022 (52 weeks)	18 years and older, males and females, *n* = 8/6	-
Pompe	AAV	Liver-specific promoter	hGAA	SPK-3006	Intravenous dose escalation	NCT04093349 (RESOLUTE) Spark Therapeutics	Phase ½ Recruiting	2020–2023 (52 weeks)	18 years and older, males and females, *n* = 20/same as current	-
Pompe	AAV8	Hybrid liver/desmin promoter	hGAA	AT845	Intravenous 2 doses	NCT04174105 (FORTIS) Audentes Therapeutics	Phase ½ Recruiting	2020–2027 (5 years)	18 to 80 years, males and females, *n* = 8/same as current	-
Danon	AAV9	CAG	hLAMP2B	RP-A501	Intravenous 2 doses	NCT03882437 Rocket Pharmaceuticals Inc.	Phase 1 recruiting	2019–2023 (3 years)	8 years to 14 years and 15 years and older, males, *n* = 24/same as current	-
LGMD2E	scAAV rh74	MHCK7	SGCB	SRP-9003	Intravenous 5 × 10^13^ vg/kg	NCT03652259 Sarepta Therapeutics, Inc.	Phase ½ active, not recruiting	2018–2020 (3 years)	4 to 15 years, males and females, *n* = 6/9	Elevated liver enzymes associated with transient increase in bilirubin (*n* = 1) [190]
Batten disease	AAV2	CU	hCLN2	-	CNS administration 3 × 10^12^ vg	NCT00151216 Weill Medical College of Cornell University/Nathan’s Battle Foundation	Phase 1 completed	2004–2019 (18 months)	3 to 18 years, males and females, *n* = 10/11	-
Batten disease	AAVrh.10	CU	hCLN2	-	Direct CNS administration 9 × 10^11^ vg/2.85 × 10^11^ vg	NCT01414985 Weill Medical College of Cornell University	Phase ½ completed	2010–2017 (18 months)	3 to 18 years, males and females, *n* = 8/16	-
Batten disease	AAVrh.10	CU	hCLN2	-	Direct CNS administration 9 × 10^11^ vg 2.85 × 10^11^ vg	NCT01161576 Weill Medical College of Cornell University/National Institute of Health	Phase 1 active, not recruiting	2010–2032 (18 months)	2 to 18 years, males and females, *n* = 25/16	-
Batten disease	scAAV9	CB	CLN6	AT-GTX-501	Intrathecal	NCT02725580 Amicus Therapeutics	Phase 1/2A active, not recruiting	2016–2021 (24 months)	1 year and older, males and females, *n* = 13/6	-
Batten disease	scAAV9	P546	CLN3	AT-GTX-502	Intrathecal 2 doses	NCT03770572 Amicus Therapeutics	Phase 1/2A active, not recruiting	2018–2023 (36 months)	3 to 10 years, males and females, *n* = 7/same as current	-
GSD1a	AAV8	Native promoter	G6Pase	DTX401	Intravenous 3 doses	NCT03517085 Ultragenyx Pharmaceutical INC	Phase 1/2 recruiting	2018–2020 (52 weeks)	18 years and older, males and females, *n* = 18/9	No treatment-related serious adverse events reported to date

DMD: Duchenne muscular dystrophy; SMA: Spinal muscular atrophy; XLMTM: X-linked myotubular myopathy; LGMD2E: Limb girdle muscular dystrophy type 2E; GSD1a: Glycogen storage disease type 1a.

**Table 2:** Preclinical animal studies in mice models of SMA.

Reference.	Promoter	Codon Optimisation	Dose vg/per Mouse	Dose vg/kg of Body Weight	Expression in CNS	Mean Survival (Days)	Adverse Events
[196]	PGK	Yes	4.5 × 10^10^	3 × 10^13^	SC: 80–140% of WT levels Brain: low	160 d (in 100% mice)	- Hyperactivity - Tail necrosis - Ear necrosis - Bilateral cataract
[194]	CBA	No	5 × 10^11^	3.3 × 10^14^	SC: 42% of WT levels	>250 d (*n* = 4, 1 death at d 97)	- Necrotic pinna
[197]	CMV	Yes	1 × 10^11^	6.7 × 10^13^	Lumbar SC: 66.5% MN Thoracic SC: 45% MN Cervical SC: 55% MN	69 d (in 80% of mice)	- Short tail - Ear necrosis - Moderate eyelid inflammation

All mice were from the SMN delta 7 strain (SMN2^+/+^, SMNΔ7^+/+^, smn^−/−^) and received an intravenous injection in the facial vein at p1 of an AAV9 solution. The doses expressed in vg/kg were calculated using a mouse body weight at p1 of 1.5 g (the average weight of a p1 SMN-Delta7 pup [193]).

**Table 3:** Micro-dystrophin clinical trials in DMD patients. NSAA: NorthStar Ambulatory Assessment score, SM: Skeletal muscle.

Trial Promoter/Product Name/Reference	Vector	Promoter	Micro-Dystrophin Domains	Dose vg/kg of Body Weight	Expression in SM	NSAA	Serious Adverse Events
Sarepta SRP-9001-101 [177]	AAVrh74	MHCK7 (SM and cardiac)	coΔR4-R23/ΔCT	2 × 10^14^	95.8% of normal	5.5 points increase after 1 year	
Pfizer PF-06939926	AAV9	Human muscle specific	-	1 × 10^14^ 3 × 10^14^	23.6% of normal 29.5% of normal	2 points increase after 1 year	In 1 patient at 3 × 10^14^ vg/kg: complement activation, acute kidney failure, thrombocytopenia
Solid SGT-001	AAV9	CK8	ΔR2-R15/ΔR18-R22/ΔCT	2 × 10^14^ 2 doses			Complement activation, acute kidney failure, thrombocytopenia (2 SAEs in 6th patient)

**Table 4:** Next-generation recombinant AAVs for improved muscle targeting, reduced liver targeting and/or a reduced immune response.

Reference	AAV Name	Parental AAV	Method	Compared with	Receptor	Muscle Transduction	Heart Transduction	Liver Transduction	Immune Response
[253]	AAV2i8	AAV2	Rational design: replacement of receptor-binding hexapeptide with corresponding residue in AAV8	AAV2/AAV8	Not HS	=AAV8 >AAV2	=AAV8 >AAV2	<AAV2 and AAV8 (40-fold lower)	Lower cross reactivity to AAV2 antibody
[257]				AAV9 (rhesus monkey)		<AAV9 (122-fold lower)	<AAV9 (46-fold lower)	<AAV9 (11-fold lower)	ND
[256]	AAV2i8G9	AAV2i8	Rational design: graft galactose-binding footprint of AAV9 in VP3 AAV2i8	AAV2i8/AAV9	Not HS Glycan	>AAV2i8>AAV9	AAV2i8 < AAV2i8G9 < AAV9	AAV2i8 < AAV2i8G9 < AAV9 (5-fold lower)	ND
[146]	AAV2.5	AAV2	Rational design: AAV2 capsid with 5 mutations from AAV1	AAV2	HS	>AAV2 (2- to 5-fold)	NA	NA	-No cellular immune response to capsid. -Lower cross-reaction to AAV2 NAb
[254]	AAV2 587 MTP	AAV2	Rational design: insertion of muscle-targeting peptide in AAV2 capsid	AAV2	Not heparin	≥AAV2 (2-fold)	>AAV2 (7-fold)	<AAV2 (2.5-fold)	ND
[258]	AAV9.45	AAV9	Directed evolution: random mutagenesis of surface-exposed regions of AAV9	AAV9	ND	≈AAV9	≈AAV9	<AAV9 (10- to 25-fold lower)	ND
[259]	AAV po1	NA	Natural pig isolate	AAV9	ND	<AAV9 (≈2–4-fold)	<AAV9 (≈3-fold)	<AAV9 (≈140-fold)	ND
				AAV5	ND	>AAV5 (1.5-fold)	<AAV5 (30-fold)	<AAV5 (≈125-fold)	No pre-existing immunity No cross-neutralisation by antisera against all common AAVs
[261]	AAV9-Y731F AAV1-Y445F/Y731F	AAV9 AAV1	Rational design: tyrosine mutations	Other mutants (no AAV of reference)	ND	Skeletal muscle > heart ≈ liver liver (3–10-fold lower) < skeletal muscle (3–10-fold lower) < heart			ND
[255]	AAV2-VNSTRLP	AAV2	Directed evolution: from AAV2 display peptide library with in vitro selection for heart tropism	AAV2 AAV9	ND	≈AAV2 <AAV9	>AAV2 (>10-fold) <AAV9	<AAV2 (≈10-fold) <AAV9	ND
[263]	AAVM41	AAV1/6/7/8	Directed evolution by shuffling the capsids of AAV1 to AAV9 and in vivo selection on skeletal muscle	AAV9 AAV6	ND	<AAV9 >AAV6	≈AAV9 >AAV6 (up to 13-fold)	<AAV9 <AAV6	Lower cross reactivity
[260]	AAVB1	AAV8 AAVrh43 (mostly)	Directed evolution of DNA shuffled library and selection on brain tissues	AAV9	Not SA Not Galactose	>AAV9 (10- to 26-fold higher)	>AAV9 (14-fold higher)	<AAV9 (3.6-fold lower)	Modestly more resistant to neutralisation than AAV9
[252]	AAVC4 AAVC7 AAVG4	NA	Ancestral reconstruction from NHP and human AAV by combinatorial variation of 32 amino acids and selection on muscle cells	AAV1	Not SA Not galactose Not HS	>AAV1 (10–31-fold higher)	NA	NA	Not resistant to neutralisation with IVIG
[265]	AAV10HB	NA	Isolation from bat faecal and intestinal tissues	AAV2 AAV8	ND	Ratio muscle/liver = 8.8 Ratio muscle/liver < 1 for AAV2 and AAV8			Reduced neutralisation with IVIG ≈AAV2
[266]	Several variants: r2.4/r2.15	AAV2	Directed evolution: random mutagenesis and selection of heparin binding or neutralising serum binding	AAV2	Not heparin	ND	ND	ND	Reduced neutralisation with serum/AAV2
[268]	Mus12	Capsid shuffled library	Directed evolution: shuffled library selected on patients’ sera and amplified in vivo in mouse muscle	AAV1/AAV2/AAV2.5/AAV6/AAV8/AAV9	ND	IM ≈ AAV6 ≈ AAV9 IV < AAV9	ND	ND	Immune escape
[267]	CAM130	AAV1	Directed evolution: rational mutagenesis on AAV1 capsid residues in contact with antibodies, library generation and evolution on vascular endothelial cells	AAV1	ND	ND	>AAV1 (2-fold)	=AAV1	Neutralisation escape to murine, NHP and human sera

IVIG: intravenous human IgG; HS: heparan sulfate; SA: sialic acid; NA: not applicable; ND: not determined.

## Abbreviations

NHP: non-human primate; SMA: spinal muscular atrophy; DMD: Duchenne myopathy disease; XLMTM: X-linked myotubular myopathy; CNS: central nervous system; AAV: adeno-associated virus; SMN: survival motor neuron; FDA: Food and Drug Administration; EMA: European Medicines Agency; SAE: serious-adverse event; miR: microRNA; PMO: phosphorodiamidate morpholino oligomer; ITR: inverted-terminal-repeat; LGMD: limb-girdle muscular dystrophy; NAbs: neutralizing antibodies; Sk-CRMs: skeletal muscle-specific cis-regulatory modules; WPRE: woodchuck hepatitis virus post-transcriptional regulatory element; APC: antigen presenting cells; sc: self-complementary.

## References

[1] Sajer S., Guardiero G.S., Scicchitano B.M. (2018). Myokines in Home-Based Functional Electrical Stimulation-Induced Recovery of Skeletal Muscle in Elderly and Permanent Denervation. Eur. J. Transl. Myol..

[2] Kern H., Carraro U. (2020). Home-Based Functional Electrical Stimulation of Human Permanent Denervated Muscles: A Narrative Review on Diagnostics, Managements, Results and Byproducts Revisited 2020. Diagnostics.

[3] Carraro U. (2020). Thirty years of translational research in Mobility Medicine: Collection of abstracts of the 2020 Padua Muscle Days. Eur. J. Transl. Myol..

[4] Sarabon N., Kozinc Z., Lofler S., Hofer C. (2020). Resistance Exercise, Electrical Muscle Stimulation, and Whole-Body Vibration in Older Adults: Systematic Review and Meta-Analysis of Randomized Controlled Trials. J. Clin. Med..

[5] Benarroch L., Bonne G., Rivier F., Hamroun D. (2019). The 2020 version of the gene table of neuromuscular disorders (nuclear genome). Neuromuscul. Disord. NMD.

[6] Kraker J., Zivkovic S.A. (2011). Autoimmune neuromuscular disorders. Curr. Neuropharmacol..

[7] Herbelet S., Rodenbach A., Paepe B., De Bleecker J.L. (2020). Anti-Inflammatory and General Glucocorticoid Physiology in Skeletal Muscles Affected by Duchenne Muscular Dystrophy: Exploration of Steroid-Sparing Agents. Int. J. Mol. Sci..

[8] Goto M., Komaki H., Takeshita E., Abe Y., Ishiyama A., Sugai K., Sasaki M., Goto Y., Nonaka I. (2016). Long-term outcomes of steroid therapy for Duchenne muscular dystrophy in Japan. Brain Dev..

[9] Biggar W.D., Harris V.A., Eliasoph L., Alman B. (2006). Long-term benefits of deflazacort treatment for boys with Duchenne muscular dystrophy in their second decade. Neuromuscul. Disord. NMD.

[10] Martiniuk F., Mehler M., Pellicer A., Tzall S., La Badie G., Hobart C., Ellenbogen A., Hirschhorn R. (1986). Isolation of a cDNA for human acid alpha-glucosidase and detection of genetic heterogeneity for mRNA in three alpha-glucosidase-deficient patients. Proc. Natl. Acad. Sci. USA.

[11] FDA (2006). Myozyme Approval Letter. https://www.accessdata.fda.gov/drugsatfda_docs/nda/2006/125141s0000_Myozyme_Approv.pdf.

[12] EMA (2014). Myozyme Alglucosidase Alfa. https://www.ema.europa.eu/en/documents/overview/myozyme-epar-summary-public_en.pdf.

[13] Kishnani P.S., Corzo D., Nicolino M., Byrne B., Mandel H., Hwu W.L., Leslie N., Levine J., Spencer C., McDonald M. (2007). Recombinant human acid [alpha]-glucosidase: Major clinical benefits in infantile-onset Pompe disease. Neurology.

[14] Nicolino M., Byrne B., Wraith J.E., Leslie N., Mandel H., Freyer D.R., Arnold G.L., Pivnick E.K., Ottinger C.J., Robinson P.H. (2009). Clinical outcomes after long-term treatment with alglucosidase alfa in infants and children with advanced Pompe disease. Genet. Med. Off. J. Am. Coll. Med. Genet..

[15] van der Ploeg A.T., Clemens P.R., Corzo D., Escolar D.M., Florence J., Groeneveld G.J., Herson S., Kishnani P.S., Laforet P., Lake S.L. (2010). A randomized study of alglucosidase alfa in late-onset Pompe’s disease. N. Engl. J. Med..

[16] Hahn S.H., Kronn D., Leslie N.D., Pena L.D.M., Tanpaiboon P., Gambello M.J., Gibson J.B., Hillman R., Stockton D.W., Day J.W. (2018). Efficacy, safety profile, and immunogenicity of alglucosidase alfa produced at the 4,000-liter scale in US children and adolescents with Pompe disease: ADVANCE, a phase IV, open-label, prospective study. Genet. Med. Off. J. Am. Coll. Med. Genet..

[17] FDA (2016). FDA Grants Accelerated Approval to First Drug for Duchenne Muscular Dystrophy. https://www.fda.gov/news-events/press-announcements/fda-grants-accelerated-approval-first-drug-duchenne-muscular-dystrophy.

[18] Koenig M., Hoffman E.P., Bertelson C.J., Monaco A.P., Feener C., Kunkel L.M. (1987). Complete cloning of the Duchenne muscular dystrophy (DMD) cDNA and preliminary genomic organization of the DMD gene in normal and affected individuals. Cell.

[19] England S.B., Nicholson L.V., Johnson M.A., Forrest S.M., Love D.R., Zubrzycka-Gaarn E.E., Bulman D.E., Harris J.B., Davies K.E. (1990). Very mild muscular dystrophy associated with the deletion of 46% of dystrophin. Nature.

[20] Charleston J.S., Schnell F.J., Dworzak J., Donoghue C., Lewis S., Chen L., Young G.D., Milici A.J., Voss J., DeAlwis U. (2018). Eteplirsen treatment for Duchenne muscular dystrophy: Exon skipping and dystrophin production. Neurology.

[21] Mendell J.R., Rodino-Klapac L.R., Sahenk Z., Roush K., Bird L., Lowes L.P., Alfano L., Gomez A.M., Lewis S., Kota J. (2013). Eteplirsen for the treatment of Duchenne muscular dystrophy. Ann. Neurol..

[22] Mendell J.R., Goemans N., Lowes L.P., Alfano L.N., Berry K., Shao J., Kaye E.M., Mercuri E., Eteplirsen Study G., Telethon Foundation D.M.D.I.N. (2016). Longitudinal effect of eteplirsen versus historical control on ambulation in Duchenne muscular dystrophy. Ann. Neurol..

[23] Alfano L.N., Charleston J.S., Connolly A.M., Cripe L., Donoghue C., Dracker R., Dworzak J., Eliopoulos H., Frank D.E., Lewis S. (2019). Long-term treatment with eteplirsen in nonambulatory patients with Duchenne muscular dystrophy. Medicine.

[24] Kinane T.B., Mayer O.H., Duda P.W., Lowes L.P., Moody S.L., Mendell J.R. (2018). Long-Term Pulmonary Function in Duchenne Muscular Dystrophy: Comparison of Eteplirsen-Treated Patients to Natural History. J. Neuromuscul. Dis..

[25] Khan N., Eliopoulos H., Han L., Kinane T.B., Lowes L.P., Mendell J.R., Gordish-Dressman H., Henricson E.K., McDonald C.M., Eteplirsen I. (2019). Eteplirsen Treatment Attenuates Respiratory Decline in Ambulatory and Non-Ambulatory Patients with Duchenne Muscular Dystrophy. J. Neuromuscul. Dis..

[26] Bushby K., Finkel R., Wong B., Barohn R., Campbell C., Comi G.P., Connolly A.M., Day J.W., Flanigan K.M., Goemans N. (2014). Ataluren treatment of patients with nonsense mutation dystrophinopathy. Muscle Nerve.

[27] McDonald C.M., Campbell C., Torricelli R.E., Finkel R.S., Flanigan K.M., Goemans N., Heydemann P., Kaminska A., Kirschner J., Muntoni F. (2017). Ataluren in patients with nonsense mutation Duchenne muscular dystrophy (ACT DMD): A multicentre, randomised, double-blind, placebo-controlled, phase 3 trial. Lancet.

[28] Mercuri E., Muntoni F., Osorio A.N., Tulinius M., Buccella F., Morgenroth L.P., Gordish-Dressman H., Jiang J., Trifillis P., Zhu J. (2020). Safety and effectiveness of ataluren: Comparison of results from the STRIDE Registry and CINRG DMD Natural History Study. J. Comp. Eff. Res..

[29] FDA (2016). FDA Approves First Drug for Spinal Muscular Atrophy. https://www.fda.gov/news-events/press-announcements/fda-approves-first-drug-spinal-muscular-atrophy.

[30] EMA (2017). First Medicine for Spinal Muscular Atrophy. https://www.ema.europa.eu/en/news/first-medicine-spinal-muscular-atrophy.

[31] Lefebvre S., Burglen L., Reboullet S., Clermont O., Burlet P., Viollet L., Benichou B., Cruaud C., Millasseau P., Zeviani M. (1995). Identification and characterization of a spinal muscular atrophy-determining gene. Cell.

[32] Mercuri E., Darras B.T., Chiriboga C.A., Day J.W., Campbell C., Connolly A.M., Iannaccone S.T., Kirschner J., Kuntz N.L., Saito K. (2018). Nusinersen versus Sham Control in Later-Onset Spinal Muscular Atrophy. N. Engl. J. Med..

[33] De Vivo D.C., Bertini E., Swoboda K.J., Hwu W.L., Crawford T.O., Finkel R.S., Kirschner J., Kuntz N.L., Parsons J.A., Ryan M.M. (2019). Nusinersen initiated in infants during the presymptomatic stage of spinal muscular atrophy: Interim efficacy and safety results from the Phase 2 NURTURE study. Neuromuscul. Disord. NMD.

[34] FDA (2020). FDA Approves Oral Treatment for Spinal Muscular Atrophy. https://www.fda.gov/news-events/press-announcements/fda-approves-oral-treatment-spinal-muscular-atrophy.

[35] Poirier A., Weetall M., Heinig K., Bucheli F., Schoenlein K., Alsenz J., Bassett S., Ullah M., Senn C., Ratni H. (2018). Risdiplam distributes and increases SMN protein in both the central nervous system and peripheral organs. Pharmacol. Res. Perspect..

[36] Ratni H., Ebeling M., Baird J., Bendels S., Bylund J., Chen K.S., Denk N., Feng Z., Green L., Guerard M. (2018). Discovery of Risdiplam, a Selective Survival of Motor Neuron-2 (SMN2) Gene Splicing Modifier for the Treatment of Spinal Muscular Atrophy (SMA). J. Med. Chem..

[37] Sturm S., Gunther A., Jaber B., Jordan P., Al Kotbi N., Parkar N., Cleary Y., Frances N., Bergauer T., Heinig K. (2019). A phase 1 healthy male volunteer single escalating dose study of the pharmacokinetics and pharmacodynamics of risdiplam (RG7916, RO7034067), a SMN2 splicing modifier. Br. J. Clin. Pharmacol..

[38] Gaudet D., Methot J., Dery S., Brisson D., Essiembre C., Tremblay G., Tremblay K., de Wal J., Twisk J., van den Bulk N. (2013). Efficacy and long-term safety of alipogene tiparvovec (AAV1-LPLS447X) gene therapy for lipoprotein lipase deficiency: An open-label trial. Gene Ther..

[39] Yla-Herttuala S. (2012). Endgame: Glybera finally recommended for approval as the first gene therapy drug in the European union. Mol. Ther. J. Am. Soc. Gene Ther..

[40] Russell S., Bennett J., Wellman J.A., Chung D.C., Yu Z.F., Tillman A., Wittes J., Pappas J., Elci O., McCague S. (2017). Efficacy and safety of voretigene neparvovec (AAV2-hRPE65v2) in patients with RPE65-mediated inherited retinal dystrophy: A randomised, controlled, open-label, phase 3 trial. Lancet.

[41] FDA (2017). FDA Approves Novel Gene Therapy to Treat Patients with a Rare Form of Inherited Vision Loss. https://www.fda.gov/news-events/press-announcements/fda-approves-novel-gene-therapy-treat-patients-rare-form-inherited-vision-loss.

[42] EMA (2018). New Gene Therapy for Rare Inherited Disorder Causing Vision Loss Recommended for Approval. https://www.ema.europa.eu/en/documents/press-release/new-gene-therapy-rare-inherited-disorder-causing-vision-loss-recommended-approval_en.pdf.

[43] FDA (2019). FDA Approves Innovative Gene Therapy to Treat Pediatric Patients with Spinal Muscular Atrophy, a Rare Disease and Leading Genetic Cause of Infant Mortality. https://www.fda.gov/news-events/press-announcements/fda-approves-innovative-gene-therapy-treat-pediatric-patients-spinal-muscular-atrophy-rare-disease.

[44] Mendell J.R., Al-Zaidy S., Shell R., Arnold W.D., Rodino-Klapac L.R., Prior T.W., Lowes L., Alfano L., Berry K., Church K. (2017). Single-Dose Gene-Replacement Therapy for Spinal Muscular Atrophy. N. Engl. J. Med..

[45] EMA (2020). New Gene Therapy to Treat Spinal Muscular Atrophy (Corrected). https://www.ema.europa.eu/en/news/new-gene-therapy-treat-spinal-muscular-atrophy-corrected.

[46] Sonntag F., Schmidt K., Kleinschmidt J.A. (2010). A viral assembly factor promotes AAV2 capsid formation in the nucleolus. Proc. Natl. Acad. Sci. USA.

[47] Ogden P.J., Kelsic E.D., Sinai S., Church G.M. (2019). Comprehensive AAV capsid fitness landscape reveals a viral gene and enables machine-guided design. Science.

[48] Russell D.W., Miller A.D., Alexander I.E. (1994). Adeno-associated virus vectors preferentially transduce cells in S phase. Proc. Natl. Acad. Sci. USA.

[49] Atchison R.W., Casto B.C., Hammon W.M. (1965). Adenovirus-Associated Defective Virus Particles. Science.

[50] Hoggan M.D., Blacklow N.R., Rowe W.P. (1966). Studies of small DNA viruses found in various adenovirus preparations: Physical, biological, and immunological characteristics. Proc. Natl. Acad. Sci. USA.

[51] Parks W.P., Green M., Pina M., Melnick J.L. (1967). Physicochemical characterization of adeno-associated satellite virus type 4 and its nucleic acid. J. Virol..

[52] Bantel-Schaal U., zur Hausen H. (1984). Characterization of the DNA of a defective human parvovirus isolated from a genital site. Virology.

[53] Rutledge E.A., Halbert C.L., Russell D.W. (1998). Infectious clones and vectors derived from adeno-associated virus (AAV) serotypes other than AAV type 2. J. Virol..

[54] Gao G.P., Alvira M.R., Wang L., Calcedo R., Johnston J., Wilson J.M. (2002). Novel adeno-associated viruses from rhesus monkeys as vectors for human gene therapy. Proc. Natl. Acad. Sci. USA.

[55] Gao G., Vandenberghe L.H., Alvira M.R., Lu Y., Calcedo R., Zhou X., Wilson J.M. (2004). Clades of Adeno-associated viruses are widely disseminated in human tissues. J. Virol..

[56] Mori S., Wang L., Takeuchi T., Kanda T. (2004). Two novel adeno-associated viruses from cynomolgus monkey: Pseudotyping characterization of capsid protein. Virology.

[57] Schmidt M., Voutetakis A., Afione S., Zheng C., Mandikian D., Chiorini J.A. (2008). Adeno-associated virus type 12 (AAV12): A novel AAV serotype with sialic acid- and heparan sulfate proteoglycan-independent transduction activity. J. Virol..

[58] Schmidt M., Govindasamy L., Afione S., Kaludov N., Agbandje-McKenna M., Chiorini J.A. (2008). Molecular characterization of the heparin-dependent transduction domain on the capsid of a novel adeno-associated virus isolate, AAV(VR-942). J. Virol..

[59] Gao G., Vandenberghe L.H., Wilson J.M. (2005). New recombinant serotypes of AAV vectors. Curr. Gene Ther..

[60] Gao G., Zhong L., Danos O. (2011). Exploiting natural diversity of AAV for the design of vectors with novel properties. Methods Mol. Biol..

[61] DiMattia M.A., Nam H.J., Van Vliet K., Mitchell M., Bennett A., Gurda B.L., McKenna R., Olson N.H., Sinkovits R.S., Potter M. (2012). Structural insight into the unique properties of adeno-associated virus serotype 9. J. Virol..

[62] Calcedo R., Vandenberghe L.H., Gao G., Lin J., Wilson J.M. (2009). Worldwide epidemiology of neutralizing antibodies to adeno-associated viruses. J. Infect. Dis..

[63] Calcedo R., Wilson J.M. (2013). Humoral Immune Response to AAV. Front. Immunol..

[64] Boutin S., Monteilhet V., Veron P., Leborgne C., Benveniste O., Montus M.F., Masurier C. (2010). Prevalence of serum IgG and neutralizing factors against adeno-associated virus (AAV) types 1, 2, 5, 6, 8, and 9 in the healthy population: Implications for gene therapy using AAV vectors. Hum. Gene Ther..

[65] Summerford C., Samulski R.J. (1998). Membrane-associated heparan sulfate proteoglycan is a receptor for adeno-associated virus type 2 virions. J. Virol..

[66] Mietzsch M., Broecker F., Reinhardt A., Seeberger P.H., Heilbronn R. (2014). Differential adeno-associated virus serotype-specific interaction patterns with synthetic heparins and other glycans. J. Virol..

[67] Walters R.W., Yi S.M., Keshavjee S., Brown K.E., Welsh M.J., Chiorini J.A., Zabner J. (2001). Binding of adeno-associated virus type 5 to 2,3-linked sialic acid is required for gene transfer. J. Biol. Chem..

[68] Kaludov N., Brown K.E., Walters R.W., Zabner J., Chiorini J.A. (2001). Adeno-associated virus serotype 4 (AAV4) and AAV5 both require sialic acid binding for hemagglutination and efficient transduction but differ in sialic acid linkage specificity. J. Virol..

[69] Wu Z., Miller E., Agbandje-McKenna M., Samulski R.J. (2006). Alpha2,3 and alpha2,6 N-linked sialic acids facilitate efficient binding and transduction by adeno-associated virus types 1 and 6. J. Virol..

[70] Shen S., Bryant K.D., Brown S.M., Randell S.H., Asokan A. (2011). Terminal N-linked galactose is the primary receptor for adeno-associated virus 9. J. Biol. Chem..

[71] Bell C.L., Vandenberghe L.H., Bell P., Limberis M.P., Gao G.P., Van Vliet K., Agbandje-McKenna M., Wilson J.M. (2011). The AAV9 receptor and its modification to improve In Vivo lung gene transfer in mice. J. Clin. Investig..

[72] Bell C.L., Gurda B.L., Van Vliet K., Agbandje-McKenna M., Wilson J.M. (2012). Identification of the galactose binding domain of the adeno-associated virus serotype 9 capsid. J. Virol..

[73] Di Pasquale G., Davidson B.L., Stein C.S., Martins I., Scudiero D., Monks A., Chiorini J.A. (2003). Identification of PDGFR as a receptor for AAV-5 transduction. Nat. Med..

[74] Akache B., Grimm D., Pandey K., Yant S.R., Xu H., Kay M.A. (2006). The 37/67-kilodalton laminin receptor is a receptor for adeno-associated virus serotypes 8, 2, 3, and 9. J. Virol..

[75] Kashiwakura Y., Tamayose K., Iwabuchi K., Hirai Y., Shimada T., Matsumoto K., Nakamura T., Watanabe M., Oshimi K., Daida H. (2005). Hepatocyte growth factor receptor is a coreceptor for adeno-associated virus type 2 infection. J. Virol..

[76] Summerford C., Bartlett J.S., Samulski R.J. (1999). AlphaVbeta5 integrin: A co-receptor for adeno-associated virus type 2 infection. Nat. Med..

[77] Qing K., Mah C., Hansen J., Zhou S., Dwarki V., Srivastava A. (1999). Human fibroblast growth factor receptor 1 is a co-receptor for infection by adeno-associated virus 2. Nat. Med..

[78] Pillay S., Meyer N.L., Puschnik A.S., Davulcu O., Diep J., Ishikawa Y., Jae L.T., Wosen J.E., Nagamine C.M., Chapman M.S. (2016). Corrigendum: An essential receptor for adeno-associated virus infection. Nature.

[79] Pillay S., Meyer N.L., Puschnik A.S., Davulcu O., Diep J., Ishikawa Y., Jae L.T., Wosen J.E., Nagamine C.M., Chapman M.S. (2016). An essential receptor for adeno-associated virus infection. Nature.

[80] Xiao P.J., Samulski R.J. (2012). Cytoplasmic trafficking, endosomal escape, and perinuclear accumulation of adeno-associated virus type 2 particles are facilitated by microtubule network. J. Virol..

[81] Buller R.M., Janik J.E., Sebring E.D., Rose J.A. (1981). Herpes simplex virus types 1 and 2 completely help adenovirus-associated virus replication. J. Virol..

[82] Georg-Fries B., Biederlack S., Wolf J., zur Hausen H. (1984). Analysis of proteins, helper dependence, and seroepidemiology of a new human parvovirus. Virology.

[83] Kotin R.M., Menninger J.C., Ward D.C., Berns K.I. (1991). Mapping and direct visualization of a region-specific viral DNA integration site on chromosome 19q13-qter. Genomics.

[84] Kotin R.M., Siniscalco M., Samulski R.J., Zhu X.D., Hunter L., Laughlin C.A., McLaughlin S., Muzyczka N., Rocchi M., Berns K.I. (1990). Site-specific integration by adeno-associated virus. Proc. Natl. Acad. Sci. USA.

[85] Samulski R.J., Zhu X., Xiao X., Brook J.D., Housman D.E., Epstein N., Hunter L.A. (1991). Targeted integration of adeno-associated virus (AAV) into human chromosome 19. EMBO J..

[86] Kotin R.M., Linden R.M., Berns K.I. (1992). Characterization of a preferred site on human chromosome 19q for integration of adeno-associated virus DNA by non-homologous recombination. EMBO J..

[87] Lamartina S., Sporeno E., Fattori E., Toniatti C. (2000). Characteristics of the adeno-associated virus preintegration site in human chromosome 19: Open chromatin conformation and transcription-competent environment. J. Virol..

[88] Huser D., Gogol-Doring A., Lutter T., Weger S., Winter K., Hammer E.M., Cathomen T., Reinert K., Heilbronn R. (2010). Integration preferences of wildtype AAV-2 for consensus rep-binding sites at numerous loci in the human genome. PLoS Pathog..

[89] La Bella T., Imbeaud S., Peneau C., Mami I., Datta S., Bayard Q., Caruso S., Hirsch T.Z., Calderaro J., Morcrette G. (2020). Adeno-associated virus in the liver: Natural history and consequences in tumour development. Gut.

[90] Nault J.C., Datta S., Imbeaud S., Franconi A., Mallet M., Couchy G., Letouze E., Pilati C., Verret B., Blanc J.F. (2015). Recurrent AAV2-related insertional mutagenesis in human hepatocellular carcinomas. Nat. Genet..

[91] Zhang H.G., Wang Y.M., Xie J.F., Liang X., Hsu H.C., Zhang X., Douglas J., Curiel D.T., Mountz J.D. (2001). Recombinant adenovirus expressing adeno-associated virus cap and rep proteins supports production of high-titer recombinant adeno-associated virus. Gene Ther..

[92] Miao C.H., Snyder R.O., Schowalter D.B., Patijn G.A., Donahue B., Winther B., Kay M.A. (1998). The kinetics of rAAV integration in the liver. Nat. Genet..

[93] Nakai H., Storm T.A., Kay M.A. (2000). Recruitment of single-stranded recombinant adeno-associated virus vector genomes and intermolecular recombination are responsible for stable transduction of liver In Vivo. J. Virol..

[94] Nakai H., Yant S.R., Storm T.A., Fuess S., Meuse L., Kay M.A. (2001). Extrachromosomal recombinant adeno-associated virus vector genomes are primarily responsible for stable liver transduction In Vivo. J. Virol..

[95] Fisher K.J., Jooss K., Alston J., Yang Y., Haecker S.E., High K., Pathak R., Raper S.E., Wilson J.M. (1997). Recombinant adeno-associated virus for muscle directed gene therapy. Nat. Med..

[96] Rutledge E.A., Russell D.W. (1997). Adeno-associated virus vector integration junctions. J. Virol..

[97] Nakai H., Iwaki Y., Kay M.A., Couto L.B. (1999). Isolation of recombinant adeno-associated virus vector-cellular DNA junctions from mouse liver. J. Virol..

[98] Nakai H., Montini E., Fuess S., Storm T.A., Grompe M., Kay M.A. (2003). AAV serotype 2 vectors preferentially integrate into active genes in mice. Nat. Genet..

[99] Miller D.G., Trobridge G.D., Petek L.M., Jacobs M.A., Kaul R., Russell D.W. (2005). Large-scale analysis of adeno-associated virus vector integration sites in normal human cells. J. Virol..

[100] Nakai H., Wu X., Fuess S., Storm T.A., Munroe D., Montini E., Burgess S.M., Grompe M., Kay M.A. (2005). Large-scale molecular characterization of adeno-associated virus vector integration in mouse liver. J. Virol..

[101] Inagaki K., Lewis S.M., Wu X., Ma C., Munroe D.J., Fuess S., Storm T.A., Kay M.A., Nakai H. (2007). DNA palindromes with a modest arm length of greater, similar 20 base pairs are a significant target for recombinant adeno-associated virus vector integration in the liver, muscles, and heart in mice. J. Virol..

[102] Miller D.G., Rutledge E.A., Russell D.W. (2002). Chromosomal effects of adeno-associated virus vector integration. Nat. Genet..

[103] Yang C.C., Xiao X., Zhu X., Ansardi D.C., Epstein N.D., Frey M.R., Matera A.G., Samulski R.J. (1997). Cellular recombination pathways and viral terminal repeat hairpin structures are sufficient for adeno-associated virus integration In Vivo and in vitro. J. Virol..

[104] Chandler R.J., LaFave M.C., Varshney G.K., Trivedi N.S., Carrillo-Carrasco N., Senac J.S., Wu W., Hoffmann V., Elkahloun A.G., Burgess S.M. (2015). Vector design influences hepatic genotoxicity after adeno-associated virus gene therapy. J. Clin. Investig..

[105] Donsante A., Miller D.G., Li Y., Vogler C., Brunt E.M., Russell D.W., Sands M.S. (2007). AAV vector integration sites in mouse hepatocellular carcinoma. Science.

[106] Li H., Malani N., Hamilton S.R., Schlachterman A., Bussadori G., Edmonson S.E., Shah R., Arruda V.R., Mingozzi F., Wright J.F. (2011). Assessing the potential for AAV vector genotoxicity in a murine model. Blood.

[107] Kaplitt M.G., Leone P., Samulski R.J., Xiao X., Pfaff D.W., O’Malley K.L., During M.J. (1994). Long-term gene expression and phenotypic correction using adeno-associated virus vectors in the mammalian brain. Nat. Genet..

[108] Pruchnic R., Cao B., Peterson Z.Q., Xiao X., Li J., Samulski R.J., Epperly M., Huard J. (2000). The use of adeno-associated virus to circumvent the maturation-dependent viral transduction of muscle fibers. Hum. Gene Ther..

[109] Manno C.S., Pierce G.F., Arruda V.R., Glader B., Ragni M., Rasko J.J., Ozelo M.C., Hoots K., Blatt P., Konkle B. (2006). Successful transduction of liver in hemophilia by AAV-Factor IX and limitations imposed by the host immune response. Nat. Med..

[110] Zincarelli C., Soltys S., Rengo G., Rabinowitz J.E. (2008). Analysis of AAV serotypes 1-9 mediated gene expression and tropism in mice after systemic injection. Mol. Ther. J. Am. Soc. Gene Ther..

[111] Sanftner L.M., Sommer J.M., Suzuki B.M., Smith P.H., Vijay S., Vargas J.A., Forsayeth J.R., Cunningham J., Bankiewicz K.S., Kao H. (2005). AAV2-mediated gene delivery to monkey putamen: Evaluation of an infusion device and delivery parameters. Exp. Neurol..

[112] Xiao W., Chirmule N., Berta S.C., McCullough B., Gao G., Wilson J.M. (1999). Gene therapy vectors based on adeno-associated virus type 1. J. Virol..

[113] Louboutin J.P., Wang L., Wilson J.M. (2005). Gene transfer into skeletal muscle using novel AAV serotypes. J. Gene Med..

[114] Riaz M., Raz Y., Moloney E.B., van Putten M., Krom Y.D., van der Maarel S.M., Verhaagen J., Raz V. (2015). Differential myofiber-type transduction preference of adeno-associated virus serotypes 6 and 9. Skelet. Muscle.

[115] Ohshima S., Shin J.H., Yuasa K., Nishiyama A., Kira J., Okada T., Takeda S. (2009). Transduction efficiency and immune response associated with the administration of AAV8 vector into dog skeletal muscle. Mol. Ther. J. Am. Soc. Gene Ther..

[116] Wang Z., Zhu T., Qiao C., Zhou L., Wang B., Zhang J., Chen C., Li J., Xiao X. (2005). Adeno-associated virus serotype 8 efficiently delivers genes to muscle and heart. Nat. Biotechnol..

[117] Nakai H., Fuess S., Storm T.A., Muramatsu S., Nara Y., Kay M.A. (2005). Unrestricted hepatocyte transduction with adeno-associated virus serotype 8 vectors in mice. J. Virol..

[118] Inagaki K., Fuess S., Storm T.A., Gibson G.A., McTiernan C.F., Kay M.A., Nakai H. (2006). Robust systemic transduction with AAV9 vectors in mice: Efficient global cardiac gene transfer superior to that of AAV8. Mol. Ther. J. Am. Soc. Gene Ther..

[119] Pacak C.A., Mah C.S., Thattaliyath B.D., Conlon T.J., Lewis M.A., Cloutier D.E., Zolotukhin I., Tarantal A.F., Byrne B.J. (2006). Recombinant adeno-associated virus serotype 9 leads to preferential cardiac transduction In Vivo. Circ. Res..

[120] Bostick B., Ghosh A., Yue Y., Long C., Duan D. (2007). Systemic AAV-9 transduction in mice is influenced by animal age but not by the route of administration. Gene Ther..

[121] Pacak C.A., Sakai Y., Thattaliyath B.D., Mah C.S., Byrne B.J. (2008). Tissue specific promoters improve specificity of AAV9 mediated transgene expression following intra-vascular gene delivery in neonatal mice. Genet. Vaccines Ther..

[122] Pan X., Yue Y., Zhang K., Lostal W., Shin J.H., Duan D. (2013). Long-term robust myocardial transduction of the dog heart from a peripheral vein by adeno-associated virus serotype-8. Hum. Gene Ther..

[123] Sarkar R., Mucci M., Addya S., Tetreault R., Bellinger D.A., Nichols T.C., Kazazian H.H. (2006). Long-term efficacy of adeno-associated virus serotypes 8 and 9 in hemophilia a dogs and mice. Hum. Gene Ther..

[124] Toromanoff A., Cherel Y., Guilbaud M., Penaud-Budloo M., Snyder R.O., Haskins M.E., Deschamps J.Y., Guigand L., Podevin G., Arruda V.R. (2008). Safety and Efficacy of Regional Intravenous (RI) Versus Intramuscular (IM) Delivery of rAAV1 and rAAV8 to Nonhuman Primate Skeletal Muscle. Mol. Ther. J. Am. Soc. Gene Ther..

[125] Gregorevic P., Blankinship M.J., Allen J.M., Crawford R.W., Meuse L., Miller D.G., Russell D.W., Chamberlain J.S. (2004). Systemic delivery of genes to striated muscles using adeno-associated viral vectors. Nat. Med..

[126] Thomas G.D. (2013). Functional muscle ischemia in Duchenne and Becker muscular dystrophy. Front. Physiol..

[127] Katwal A.B., Konkalmatt P.R., Piras B.A., Hazarika S., Li S.S., John Lye R., Sanders J.M., Ferrante E.A., Yan Z., Annex B.H. (2013). Adeno-associated virus serotype 9 efficiently targets ischemic skeletal muscle following systemic delivery. Gene Ther..

[128] Arnett A.L., Konieczny P., Ramos J.N., Hall J., Odom G., Yablonka-Reuveni Z., Chamberlain J.R., Chamberlain J.S. (2014). Adeno-associated viral (AAV) vectors do not efficiently target muscle satellite cells. Mol. Ther. Methods Clin. Dev..

[129] Jiang H., Pierce G.F., Ozelo M.C., de Paula E.V., Vargas J.A., Smith P., Sommer J., Luk A., Manno C.S., High K.A. (2006). Evidence of multiyear factor IX expression by AAV-mediated gene transfer to skeletal muscle in an individual with severe hemophilia B. Mol. Ther. J. Am. Soc. Gene Ther..

[130] Bish L.T., Morine K., Sleeper M.M., Sanmiguel J., Wu D., Gao G., Wilson J.M., Sweeney H.L. (2008). Adeno-associated virus (AAV) serotype 9 provides global cardiac gene transfer superior to AAV1, AAV6, AAV7, and AAV8 in the mouse and rat. Hum. Gene Ther..

[131] Vandendriessche T., Thorrez L., Acosta-Sanchez A., Petrus I., Wang L., Ma L., De Waele L., Iwasaki Y., Gillijns V., Wilson J.M. (2007). Efficacy and safety of adeno-associated viral vectors based on serotype 8 and 9 vs. lentiviral vectors for hemophilia B gene therapy. J. Thromb. Haemost..

[132] Zincarelli C., Soltys S., Rengo G., Koch W.J., Rabinowitz J.E. (2010). Comparative cardiac gene delivery of adeno-associated virus serotypes 1-9 reveals that AAV6 mediates the most efficient transduction in mouse heart. Clin. Transl. Sci..

[133] Foust K.D., Kaspar B.K. (2009). Over the barrier and through the blood: To CNS delivery we go. Cell Cycle.

[134] Foust K.D., Nurre E., Montgomery C.L., Hernandez A., Chan C.M., Kaspar B.K. (2009). Intravascular AAV9 preferentially targets neonatal neurons and adult astrocytes. Nat. Biotechnol..

[135] Gray S.J., Matagne V., Bachaboina L., Yadav S., Ojeda S.R., Samulski R.J. (2011). Preclinical differences of intravascular AAV9 delivery to neurons and glia: A comparative study of adult mice and nonhuman primates. Mol. Ther. J. Am. Soc. Gene Ther..

[136] Samaranch L., Salegio E.A., San Sebastian W., Kells A.P., Foust K.D., Bringas J.R., Lamarre C., Forsayeth J., Kaspar B.K., Bankiewicz K.S. (2012). Adeno-associated virus serotype 9 transduction in the central nervous system of nonhuman primates. Hum. Gene Ther..

[137] Duque S., Joussemet B., Riviere C., Marais T., Dubreil L., Douar A.M., Fyfe J., Moullier P., Colle M.A., Barkats M. (2009). Intravenous administration of self-complementary AAV9 enables transgene delivery to adult motor neurons. Mol. Ther. J. Am. Soc. Gene Ther..

[138] Bevan A.K., Duque S., Foust K.D., Morales P.R., Braun L., Schmelzer L., Chan C.M., McCrate M., Chicoine L.G., Coley B.D. (2011). Systemic gene delivery in large species for targeting spinal cord, brain, and peripheral tissues for pediatric disorders. Mol. Ther. J. Am. Soc. Gene Ther..

[139] Yue Y., Pan X., Hakim C.H., Kodippili K., Zhang K., Shin J.H., Yang H.T., McDonald T., Duan D. (2015). Safe and bodywide muscle transduction in young adult Duchenne muscular dystrophy dogs with adeno-associated virus. Hum. Mol. Genet..

[140] Shin J.H., Pan X., Hakim C.H., Yang H.T., Yue Y., Zhang K., Terjung R.L., Duan D. (2013). Microdystrophin ameliorates muscular dystrophy in the canine model of duchenne muscular dystrophy. Mol. Ther. J. Am. Soc. Gene Ther..

[141] Kornegay J.N., Li J., Bogan J.R., Bogan D.J., Chen C., Zheng H., Wang B., Qiao C., Howard J.F., Xiao X. (2010). Widespread muscle expression of an AAV9 human mini-dystrophin vector after intravenous injection in neonatal dystrophin-deficient dogs. Mol. Ther. J. Am. Soc. Gene Ther..

[142] Yue Y., Ghosh A., Long C., Bostick B., Smith B.F., Kornegay J.N., Duan D. (2008). A single intravenous injection of adeno-associated virus serotype-9 leads to whole body skeletal muscle transduction in dogs. Mol. Ther. J. Am. Soc. Gene Ther..

[143] Mendell J.R., Sahenk Z., Malik V., Gomez A.M., Flanigan K.M., Lowes L.P., Alfano L.N., Berry K., Meadows E., Lewis S. (2015). A phase 1/2a follistatin gene therapy trial for becker muscular dystrophy. Mol. Ther. J. Am. Soc. Gene Ther..

[144] Mendell J.R., Sahenk Z., Al-Zaidy S., Rodino-Klapac L.R., Lowes L.P., Alfano L.N., Berry K., Miller N., Yalvac M., Dvorchik I. (2017). Follistatin Gene Therapy for Sporadic Inclusion Body Myositis Improves Functional Outcomes. Mol. Ther. J. Am. Soc. Gene Ther..

[145] Mendell J.R., Campbell K., Rodino-Klapac L., Sahenk Z., Shilling C., Lewis S., Bowles D., Gray S., Li C., Galloway G. (2010). Dystrophin immunity in Duchenne’s muscular dystrophy. N. Engl. J. Med..

[146] Bowles D.E., McPhee S.W., Li C., Gray S.J., Samulski J.J., Camp A.S., Li J., Wang B., Monahan P.E., Rabinowitz J.E. (2012). Phase 1 gene therapy for Duchenne muscular dystrophy using a translational optimized AAV vector. Mol. Ther. J. Am. Soc. Gene Ther..

[147] Smith B.K., Collins S.W., Conlon T.J., Mah C.S., Lawson L.A., Martin A.D., Fuller D.D., Cleaver B.D., Clement N., Phillips D. (2013). Phase I/II trial of adeno-associated virus-mediated alpha-glucosidase gene therapy to the diaphragm for chronic respiratory failure in Pompe disease: Initial safety and ventilatory outcomes. Hum. Gene Ther..

[148] Brooks A.R., Harkins R.N., Wang P., Qian H.S., Liu P., Rubanyi G.M. (2004). Transcriptional silencing is associated with extensive methylation of the CMV promoter following adenoviral gene delivery to muscle. J. Gene Med..

[149] Qin L., Ding Y., Pahud D.R., Chang E., Imperiale M.J., Bromberg J.S. (1997). Promoter attenuation in gene therapy: Interferon-gamma and tumor necrosis factor-alpha inhibit transgene expression. Hum. Gene Ther..

[150] Duan B., Cheng L., Gao Y., Yin F.X., Su G.H., Shen Q.Y., Liu K., Hu X., Liu X., Li G.P. (2012). Silencing of fat-1 transgene expression in sheep may result from hypermethylation of its driven cytomegalovirus (CMV) promoter. Theriogenology.

[151] Harms J.S., Splitter G.A. (1995). Interferon-gamma inhibits transgene expression driven by SV40 or CMV promoters but augments expression driven by the mammalian MHC I promoter. Hum. Gene Ther..

[152] Qin J.Y., Zhang L., Clift K.L., Hulur I., Xiang A.P., Ren B.Z., Lahn B.T. (2010). Systematic comparison of constitutive promoters and the doxycycline-inducible promoter. PLoS ONE.

[153] Al-Zaidy S., Pickard A.S., Kotha K., Alfano L.N., Lowes L., Paul G., Church K., Lehman K., Sproule D.M., Dabbous O. (2019). Health outcomes in spinal muscular atrophy type 1 following AVXS-101 gene replacement therapy. Pediatr. Pulmonol..

[154] Al-Zaidy S.A., Kolb S.J., Lowes L., Alfano L.N., Shell R., Church K.R., Nagendran S., Sproule D.M., Feltner D.E., Wells C. (2019). AVXS-101 (Onasemnogene Abeparvovec) for SMA1: Comparative Study with a Prospective Natural History Cohort. J. Neuromuscul. Dis..

[155] Dabbous O., Maru B., Jansen J.P., Lorenzi M., Cloutier M., Guerin A., Pivneva I., Wu E.Q., Arjunji R., Feltner D. (2019). Survival, Motor Function, and Motor Milestones: Comparison of AVXS-101 Relative to Nusinersen for the Treatment of Infants with Spinal Muscular Atrophy Type 1. Adv. Ther..

[156] Lowes L.P., Alfano L.N., Arnold W.D., Shell R., Prior T.W., McColly M., Lehman K.J., Church K., Sproule D.M., Nagendran S. (2019). Impact of Age and Motor Function in a Phase 1/2A Study of Infants with SMA Type 1 Receiving Single-Dose Gene Replacement Therapy. Pediatr. Neurol..

[157] Hartigan-O’Connor D., Kirk C.J., Crawford R., Mule J.J., Chamberlain J.S. (2001). Immune evasion by muscle-specific gene expression in dystrophic muscle. Mol. Ther. J. Am. Soc. Gene Ther..

[158] Yuasa K., Sakamoto M., Miyagoe-Suzuki Y., Tanouchi A., Yamamoto H., Li J., Chamberlain J.S., Xiao X., Takeda S. (2002). Adeno-associated virus vector-mediated gene transfer into dystrophin-deficient skeletal muscles evokes enhanced immune response against the transgene product. Gene Ther..

[159] Weeratna R.D., Wu T., Efler S.M., Zhang L., Davis H.L. (2001). Designing gene therapy vectors: Avoiding immune responses by using tissue-specific promoters. Gene Ther..

[160] Fabre E.E., Bigey P., Orsini C., Scherman D. (2006). Comparison of promoter region constructs for In Vivo intramuscular expression. J. Gene Med..

[161] Talbot G.E., Waddington S.N., Bales O., Tchen R.C., Antoniou M.N. (2010). Desmin-regulated lentiviral vectors for skeletal muscle gene transfer. Mol. Ther. J. Am. Soc. Gene Ther..

[162] Aikawa R., Huggins G.S., Snyder R.O. (2002). Cardiomyocyte-specific gene expression following recombinant adeno-associated viral vector transduction. J. Biol. Chem..

[163] Childers M.K., Joubert R., Poulard K., Moal C., Grange R.W., Doering J.A., Lawlor M.W., Rider B.E., Jamet T., Daniele N. (2014). Gene therapy prolongs survival and restores function in murine and canine models of myotubular myopathy. Sci. Transl. Med..

[164] Shieh P.B., Kuntz N., Smith B., Dowling J.J., Müller-Felber W., Bönnemann C.G., Servais L., Muntoni F., Blaschek A., Neuhaus S. (2020). ASPIRO Gene Therapy Trial In X-Linked Myotubular Myopathy (XLMTM): Update on Preliminary Safety And Efficacy Findings up to 72 Weeks Post-Treatment (1053). Neurology.

[165] Amacher S.L., Buskin J.N., Hauschka S.D. (1993). Multiple regulatory elements contribute differentially to muscle creatine kinase enhancer activity in skeletal and cardiac muscle. Mol. Cell. Biol..

[166] Nguyen Q.G., Buskin J.N., Himeda C.L., Fabre-Suver C., Hauschka S.D. (2003). Transgenic and tissue culture analyses of the muscle creatine kinase enhancer Trex control element in skeletal and cardiac muscle indicate differences in gene expression between muscle types. Transgenic Res..

[167] Jaynes J.B., Chamberlain J.S., Buskin J.N., Johnson J.E., Hauschka S.D. (1986). Transcriptional regulation of the muscle creatine kinase gene and regulated expression in transfected mouse myoblasts. Mol. Cell. Biol..

[168] Takeshita F., Takase K., Tozuka M., Saha S., Okuda K., Ishii N., Sasaki S. (2007). Muscle creatine kinase/SV40 hybrid promoter for muscle-targeted long-term transgene expression. Int. J. Mol. Med..

[169] Hauser M.A., Robinson A., Hartigan-O’Connor D., Williams-Gregory D.A., Buskin J.N., Apone S., Kirk C.J., Hardy S., Hauschka S.D., Chamberlain J.S. (2000). Analysis of muscle creatine kinase regulatory elements in recombinant adenoviral vectors. Mol. Ther. J. Am. Soc. Gene Ther..

[170] Wang B., Li J., Fu F.H., Chen C., Zhu X., Zhou L., Jiang X., Xiao X. (2008). Construction and analysis of compact muscle-specific promoters for AAV vectors. Gene Ther..

[171] Sahenk Z., Galloway G., Clark K.R., Malik V., Rodino-Klapac L.R., Kaspar B.K., Chen L., Braganza C., Montgomery C., Mendell J.R. (2014). AAV1.NT-3 gene therapy for charcot-marie-tooth neuropathy. Mol. Ther. J. Am. Soc. Gene Ther..

[172] Rodino-Klapac L.R., Lee J.S., Mulligan R.C., Clark K.R., Mendell J.R. (2008). Lack of toxicity of alpha-sarcoglycan overexpression supports clinical gene transfer trial in LGMD2D. Neurology.

[173] Mendell J.R., Rodino-Klapac L.R., Rosales X.Q., Coley B.D., Galloway G., Lewis S., Malik V., Shilling C., Byrne B.J., Conlon T. (2010). Sustained alpha-sarcoglycan gene expression after gene transfer in limb-girdle muscular dystrophy, type 2D. Ann. Neurol..

[174] Mendell J.R., Rodino-Klapac L.R., Rosales-Quintero X., Kota J., Coley B.D., Galloway G., Craenen J.M., Lewis S., Malik V., Shilling C. (2009). Limb-girdle muscular dystrophy type 2D gene therapy restores alpha-sarcoglycan and associated proteins. Ann. Neurol..

[175] Salva M.Z., Himeda C.L., Tai P.W., Nishiuchi E., Gregorevic P., Allen J.M., Finn E.E., Nguyen Q.G., Blankinship M.J., Meuse L. (2007). Design of tissue-specific regulatory cassettes for high-level rAAV-mediated expression in skeletal and cardiac muscle. Mol. Ther. J. Am. Soc. Gene Ther..

[176] Sun B., Young S.P., Li P., Di C., Brown T., Salva M.Z., Li S., Bird A., Yan Z., Auten R. (2008). Correction of multiple striated muscles in murine Pompe disease through adeno-associated virus-mediated gene therapy. Mol. Ther. J. Am. Soc. Gene Ther..

[177] Mendell J.R., Sahenk Z., Lehman K., Nease C., Lowes L.P., Miller N.F., Iammarino M.A., Alfano L.N., Nicholl A., Al-Zaidy S. (2020). Assessment of Systemic Delivery of rAAVrh74.MHCK7.micro-dystrophin in Children With Duchenne Muscular Dystrophy: A Nonrandomized Controlled Trial. JAMA Neurol..

[178] Li X., Eastman E.M., Schwartz R.J., Draghia-Akli R. (1999). Synthetic muscle promoters: Activities exceeding naturally occurring regulatory sequences. Nat. Biotechnol..

[179] Cordier L., Gao G.P., Hack A.A., McNally E.M., Wilson J.M., Chirmule N., Sweeney H.L. (2001). Muscle-specific promoters may be necessary for adeno-associated virus-mediated gene transfer in the treatment of muscular dystrophies. Hum. Gene Ther..

[180] Fougerousse F., Bartoli M., Poupiot J., Arandel L., Durand M., Guerchet N., Gicquel E., Danos O., Richard I. (2007). Phenotypic correction of alpha-sarcoglycan deficiency by intra-arterial injection of a muscle-specific serotype 1 rAAV vector. Mol. Ther. J. Am. Soc. Gene Ther..

[181] Ziegler R.J., Lonning S.M., Armentano D., Li C., Souza D.W., Cherry M., Ford C., Barbon C.M., Desnick R.J., Gao G. (2004). AAV2 vector harboring a liver-restricted promoter facilitates sustained expression of therapeutic levels of alpha-galactosidase A and the induction of immune tolerance in Fabry mice. Mol. Ther. J. Am. Soc. Gene Ther..

[182] Poupiot J., Costa Verdera H., Hardet R., Colella P., Collaud F., Bartolo L., Davoust J., Sanatine P., Mingozzi F., Richard I. (2019). Role of Regulatory T Cell and Effector T Cell Exhaustion in Liver-Mediated Transgene Tolerance in Muscle. Mol. Ther. Methods Clin. Dev..

[183] Bartolo L., Li Chung Tong S., Chappert P., Urbain D., Collaud F., Colella P., Richard I., Ronzitti G., Demengeot J., Gross D.A. (2019). Dual muscle-liver transduction imposes immune tolerance for muscle transgene engraftment despite preexisting immunity. JCI Insight.

[184] Colella P., Sellier P., Costa Verdera H., Puzzo F., van Wittenberghe L., Guerchet N., Daniele N., Gjata B., Marmier S., Charles S. (2019). AAV Gene Transfer with Tandem Promoter Design Prevents Anti-transgene Immunity and Provides Persistent Efficacy in Neonate Pompe Mice. Mol. Ther. Methods Clin. Dev..

[185] GlobeNewswire (2020). Solid Biosciences Provides Update Regarding SGT-001 Phase I/II Clinical Hold on IGNITE DMD. https://www.globenewswire.com/news-release/2020/05/07/2029328/0/en/Solid-Biosciences-Provides-Update-regarding-SGT-001-Phase-I-II-Clinical-Hold-on-IGNITE-DMD.html.

[186] GlobeNewswire (2018). Solid Biosciences Announces Clinical Hold On SGT-001 Phase I/II Clinical Trial for Duchenne Muscular Dystrophy. https://www.globenewswire.com/news-release/2018/03/14/1422770/0/en/Solid-Biosciences-Announces-Clinical-Hold-On-SGT-001-Phase-I-II-Clinical-Trial-For-Duchenne-Muscular-Dystrophy.html.

[187] Binks M. (2019). Early, Initial Data from C3391001, a First-In-Human Safety Study of PF-06939926, a Mini-Dystrophin Gene Therapy for the Potential Treatment of DMD.

[188] Novartis (2020). AveXis Presents AVXS-101 IT Data Demonstrating Remarkable Increases in HFMSE Scores and a Consistent Clinically Meaningful Response in Older Patients with SMA Type 2. https://www.novartis.com/news/media-releases/avexis-presents-avxs-101-it-data-demonstrating-remarkable-increases-hfmse-scores-and-consistent-clinically-meaningful-response-older-patients-sma-type-2.

[189] Pharma F. (2020). Audentes’ Gene Therapy AT132 Hit with FDA Clinical Hold after Second Patient Death. https://www.firstwordpharma.com/node/1736153.

[190] Rodino-klapac L.R., Pozsgai E.R., Lewis S., Griffin D.A., Meadows A.S., Lehman K., Church K., Lowes L., Mendel J.R. (2019). Systemic Gene Transfer with AAVrh74.MHCK7.SGCB Increased β-sarcoglycan Expression in Patients with Limb Girdle Muscular Dystrophy Type 2E. Neuropediatrics.

[191] Zhang Z., Lotti F., Dittmar K., Younis I., Wan L., Kasim M., Dreyfuss G. (2008). SMN deficiency causes tissue-specific perturbations in the repertoire of snRNAs and widespread defects in splicing. Cell.

[192] Wijngaarde C.A., Blank A.C., Stam M., Wadman R.I., van den Berg L.H., van der Pol W.L. (2017). Cardiac pathology in spinal muscular atrophy: A systematic review. Orphanet J. Rare Dis..

[193] Meyer K., Ferraiuolo L., Schmelzer L., Braun L., McGovern V., Likhite S., Michels O., Govoni A., Fitzgerald J., Morales P. (2015). Improving single injection CSF delivery of AAV9-mediated gene therapy for SMA: A dose-response study in mice and nonhuman primates. Mol. Ther. J. Am. Soc. Gene Ther..

[194] Foust K.D., Wang X., McGovern V.L., Braun L., Bevan A.K., Haidet A.M., Le T.T., Morales P.R., Rich M.M., Burghes A.H. (2010). Rescue of the spinal muscular atrophy phenotype in a mouse model by early postnatal delivery of SMN. Nat. Biotechnol..

[195] Bevan A.K., Hutchinson K.R., Foust K.D., Braun L., McGovern V.L., Schmelzer L., Ward J.G., Petruska J.C., Lucchesi P.A., Burghes A.H. (2010). Early heart failure in the SMNDelta7 model of spinal muscular atrophy and correction by postnatal scAAV9-SMN delivery. Hum. Mol. Genet..

[196] Dominguez E., Marais T., Chatauret N., Benkhelifa-Ziyyat S., Duque S., Ravassard P., Carcenac R., Astord S., Pereira de Moura A., Voit T. (2011). Intravenous scAAV9 delivery of a codon-optimized SMN1 sequence rescues SMA mice. Hum. Mol. Genet..

[197] Valori C.F., Ning K., Wyles M., Mead R.J., Grierson A.J., Shaw P.J., Azzouz M. (2010). Systemic delivery of scAAV9 expressing SMN prolongs survival in a model of spinal muscular atrophy. Sci. Transl. Med..

[198] McCarty D.M. (2008). Self-complementary AAV vectors; advances and applications. Mol. Ther. J. Am. Soc. Gene Ther..

[199] Finkel R.S., Mercuri E., Darras B.T., Connolly A.M., Kuntz N.L., Kirschner J., Chiriboga C.A., Saito K., Servais L., Tizzano E. (2017). Nusinersen versus Sham Control in Infantile-Onset Spinal Muscular Atrophy. N. Engl. J. Med..

[200] Hinderer C., Katz N., Buza E.L., Dyer C., Goode T., Bell P., Richman L.K., Wilson J.M. (2018). Severe Toxicity in Nonhuman Primates and Piglets Following High-Dose Intravenous Administration of an Adeno-Associated Virus Vector Expressing Human SMN. Hum. Gene Ther..

[201] Hordeaux J., Wang Q., Katz N., Buza E.L., Bell P., Wilson J.M. (2018). The Neurotropic Properties of AAV-PHP.B Are Limited to C57BL/6J Mice. Mol. Ther. J. Am. Soc. Gene Ther..

[202] Assinger A. (2014). Platelets and infection—An emerging role of platelets in viral infection. Front. Immunol..

[203] Harper S.Q., Hauser M.A., DelloRusso C., Duan D., Crawford R.W., Phelps S.F., Harper H.A., Robinson A.S., Engelhardt J.F., Brooks S.V. (2002). Modular flexibility of dystrophin: Implications for gene therapy of Duchenne muscular dystrophy. Nat. Med..

[204] Le Guiner C., Montus M., Servais L., Cherel Y., Francois V., Thibaud J.L., Wary C., Matot B., Larcher T., Guigand L. (2014). Forelimb treatment in a large cohort of dystrophic dogs supports delivery of a recombinant AAV for exon skipping in Duchenne patients. Mol. Ther. J. Am. Soc. Gene Ther..

[205] Goyenvalle A., Babbs A., Wright J., Wilkins V., Powell D., Garcia L., Davies K.E. (2012). Rescue of severely affected dystrophin/utrophin-deficient mice through scAAV-U7snRNA-mediated exon skipping. Hum. Mol. Genet..

[206] Goyenvalle A., Vulin A., Fougerousse F., Leturcq F., Kaplan J.C., Garcia L., Danos O. (2004). Rescue of dystrophic muscle through U7 snRNA-mediated exon skipping. Science.

[207] Vulin A., Barthelemy I., Goyenvalle A., Thibaud J.L., Beley C., Griffith G., Benchaouir R., le Hir M., Unterfinger Y., Lorain S. (2012). Muscle function recovery in golden retriever muscular dystrophy after AAV1-U7 exon skipping. Mol. Ther. J. Am. Soc. Gene Ther..

[208] Bish L.T., Sleeper M.M., Forbes S.C., Wang B., Reynolds C., Singletary G.E., Trafny D., Morine K.J., Sanmiguel J., Cecchini S. (2012). Long-term restoration of cardiac dystrophin expression in golden retriever muscular dystrophy following rAAV6-mediated exon skipping. Mol. Ther. J. Am. Soc. Gene Ther..

[209] Barbash I.M., Cecchini S., Faranesh A.Z., Virag T., Li L., Yang Y., Hoyt R.F., Kornegay J.N., Bogan J.R., Garcia L. (2013). MRI roadmap-guided transendocardial delivery of exon-skipping recombinant adeno-associated virus restores dystrophin expression in a canine model of Duchenne muscular dystrophy. Gene Ther..

[210] Gregorevic P., Allen J.M., Minami E., Blankinship M.J., Haraguchi M., Meuse L., Finn E., Adams M.E., Froehner S.C., Murry C.E. (2006). rAAV6-microdystrophin preserves muscle function and extends lifespan in severely dystrophic mice. Nat. Med..

[211] Gregorevic P., Blankinship M.J., Allen J.M., Chamberlain J.S. (2008). Systemic microdystrophin gene delivery improves skeletal muscle structure and function in old dystrophic mdx mice. Mol. Ther. J. Am. Soc. Gene Ther..

[212] Rodgers B.D., Bishaw Y., Kagel D., Ramos J.N., Maricelli J.W. (2020). Micro-dystrophin Gene Therapy Partially Enhances Exercise Capacity in Older Adult mdx Mice. Mol. Ther. Methods Clin. Dev..

[213] Rodino-Klapac L.R., Janssen P.M., Montgomery C.L., Coley B.D., Chicoine L.G., Clark K.R., Mendell J.R. (2007). A translational approach for limb vascular delivery of the micro-dystrophin gene without high volume or high pressure for treatment of Duchenne muscular dystrophy. J. Transl. Med..

[214] Rodino-Klapac L.R., Montgomery C.L., Bremer W.G., Shontz K.M., Malik V., Davis N., Sprinkle S., Campbell K.J., Sahenk Z., Clark K.R. (2010). Persistent expression of FLAG-tagged micro dystrophin in nonhuman primates following intramuscular and vascular delivery. Mol. Ther. J. Am. Soc. Gene Ther..

[215] Foster H., Sharp P.S., Athanasopoulos T., Trollet C., Graham I.R., Foster K., Wells D.J., Dickson G. (2008). Codon and mRNA sequence optimization of microdystrophin transgenes improves expression and physiological outcome in dystrophic mdx mice following AAV2/8 gene transfer. Mol. Ther. J. Am. Soc. Gene Ther..

[216] Le Guiner C., Servais L., Montus M., Larcher T., Fraysse B., Moullec S., Allais M., Francois V., Dutilleul M., Malerba A. (2017). Long-term microdystrophin gene therapy is effective in a canine model of Duchenne muscular dystrophy. Nat. Commun..

[217] Bostick B., Yue Y., Lai Y., Long C., Li D., Duan D. (2008). Adeno-associated virus serotype-9 microdystrophin gene therapy ameliorates electrocardiographic abnormalities in mdx mice. Hum. Gene Ther..

[218] Hakim C.H., Wasala N.B., Pan X., Kodippili K., Yue Y., Zhang K., Yao G., Haffner B., Duan S.X., Ramos J. (2017). A Five-Repeat Micro-Dystrophin Gene Ameliorated Dystrophic Phenotype in the Severe DBA/2J-mdx Model of Duchenne Muscular Dystrophy. Mol. Ther. Methods Clin. Dev..

[219] Bostick B., Shin J.H., Yue Y., Duan D. (2011). AAV-microdystrophin therapy improves cardiac performance in aged female mdx mice. Mol. Ther. J. Am. Soc. Gene Ther..

[220] Shin J.H., Nitahara-Kasahara Y., Hayashita-Kinoh H., Ohshima-Hosoyama S., Kinoshita K., Chiyo T., Okada H., Okada T., Takeda S. (2011). Improvement of cardiac fibrosis in dystrophic mice by rAAV9-mediated microdystrophin transduction. Gene Ther..

[221] Hakim C.H., Clement N., Wasala L.P., Yang H.T., Yue Y., Zhang K., Kodippili K., Adamson-Small L., Pan X., Schneider J.S. (2020). Micro-dystrophin AAV Vectors Made by Transient Transfection and Herpesvirus System Are Equally Potent in Treating mdx Mouse Muscle Disease. Mol. Ther. Methods Clin. Dev..

[222] Lai Y., Thomas G.D., Yue Y., Yang H.T., Li D., Long C., Judge L., Bostick B., Chamberlain J.S., Terjung R.L. (2009). Dystrophins carrying spectrin-like repeats 16 and 17 anchor nNOS to the sarcolemma and enhance exercise performance in a mouse model of muscular dystrophy. J. Clin. Investig..

[223] Griffin D.A., Potter R.A., Pozsgai E.R., Peterson E.L., Rodino-Klapac L.R. (2019). Adeno-Associated Virus Serotype rh74 Prevalence in Muscular Dystrophy Population. Mol. Ther..

[224] Zygmunt D.A., Crowe K.E., Flanigan K.M., Martin P.T. (2017). Comparison of Serum rAAV Serotype-Specific Antibodies in Patients with Duchenne Muscular Dystrophy, Becker Muscular Dystrophy, Inclusion Body Myositis, or GNE Myopathy. Hum. Gene Ther..

[225] Fu H., Meadows A.S., Pineda R.J., Kunkler K.L., Truxal K.V., McBride K.L., Flanigan K.M., McCarty D.M. (2017). Differential Prevalence of Antibodies Against Adeno-Associated Virus in Healthy Children and Patients with Mucopolysaccharidosis III: Perspective for AAV-Mediated Gene Therapy. Hum. Gene Ther. Clin. Dev..

[226] Potter R.A., Griffin D.A., Heller K.N., Peterson E.L., Clark E.K., Mendell J.R., Rodino-Klapac L.R. Dose-escalation study of systematically delivered rAAVrh74.MHCK7.micro-dystrophin in the mdx mouse model of Duchenne muscular dystrophy. Proceedings of the American Society of Gene and Cell Therapy Annual Meeting.

[227] Potter R.A., Griffin D.A., Heller K.N., Peterson E.L., Johnson R.W., Clark E.K., Mendell J.R., Rodino-Klapac L.R. (2018). Dose Escalation Study of Systemically Delivered AAVrh74.MHCK7.Micro-Dystrophin in the Mdx Mouse Model of DMD. Mol. Ther..

[228] Mendell J.R., Chicoine L.G., Al-Zaidy S.A., Sahenk Z., Lehman K., Lowes L., Miller N., Alfano L., Galliers B., Lewis S. (2019). Gene Delivery for Limb-Girdle Muscular Dystrophy Type 2D by Isolated Limb Infusion. Hum. Gene Ther..

[229] Asher D.R., Thapa K., Dharia S.D., Khan N., Potter R.A., Rodino-Klapac L.R., Mendell J.R. (2020). Clinical development on the frontier: Gene therapy for duchenne muscular dystrophy. Expert Opin. Biol. Ther..

[230] Nathwani A.C., Tuddenham E.G., Rangarajan S., Rosales C., McIntosh J., Linch D.C., Chowdary P., Riddell A., Pie A.J., Harrington C. (2011). Adenovirus-associated virus vector-mediated gene transfer in hemophilia B. N. Engl. J. Med..

[231] George L.A., Sullivan S.K., Giermasz A., Rasko J.E.J., Samelson-Jones B.J., Ducore J., Cuker A., Sullivan L.M., Majumdar S., Teitel J. (2017). Hemophilia B Gene Therapy with a High-Specific-Activity Factor IX Variant. N. Engl. J. Med..

[232] Solid Biosciences (2018). Letter to the Duchenne Community about the Status of the IGNITE DMD Clinical Trial. https://www.solidbio.com/about/media/news/letter-to-the-duchenne-community-about-the-status-of-the-ignite-dmd-clinical-trial.

[233] Solid Biosciences (2020). Solid Biosciences Announces FDA Lifts Clinical Hold on IGNITE DMD Clinical Trial. https://www.solidbio.com/about/media/press-releases/solid-biosciences-announces-fda-lifts-clinical-hold-on-ignite-dmd-clinical-trial.

[234] Hakim C.H., Kodippili K., Jenkins G., Yang H., Pan X., Lessa T., Leach S., Emter C., Yue Y., Zhang K. (2018). AAV micro-dystrophin therapy ameliorates muscular dystrophy in young adult Duchenne muscular dystrophy dogs for up to thirty months following injection. Mol. Ther..

[235] Simon A.K., Hollander G.A., McMichael A. (2015). Evolution of the immune system in humans from infancy to old age. Proc. Biol. Sci..

[236] Laporte J., Hu L.J., Kretz C., Mandel J.L., Kioschis P., Coy J.F., Klauck S.M., Poustka A., Dahl N. (1996). A gene mutated in X-linked myotubular myopathy defines a new putative tyrosine phosphatase family conserved in yeast. Nat. Genet..

[237] Blondeau F., Laporte J., Bodin S., Superti-Furga G., Payrastre B., Mandel J.L. (2000). Myotubularin, a phosphatase deficient in myotubular myopathy, acts on phosphatidylinositol 3-kinase and phosphatidylinositol 3-phosphate pathway. Hum. Mol. Genet..

[238] Taylor G.S., Maehama T., Dixon J.E. (2000). Myotubularin, a protein tyrosine phosphatase mutated in myotubular myopathy, dephosphorylates the lipid second messenger, phosphatidylinositol 3-phosphate. Proc. Natl. Acad. Sci. USA.

[239] Buj-Bello A., Laugel V., Messaddeq N., Zahreddine H., Laporte J., Pellissier J.F., Mandel J.L. (2002). The lipid phosphatase myotubularin is essential for skeletal muscle maintenance but not for myogenesis in mice. Proc. Natl. Acad. Sci. USA.

[240] Beggs A.H., Bohm J., Snead E., Kozlowski M., Maurer M., Minor K., Childers M.K., Taylor S.M., Hitte C., Mickelson J.R. (2010). MTM1 mutation associated with X-linked myotubular myopathy in Labrador Retrievers. Proc. Natl. Acad. Sci. USA.

[241] Grange R.W., Doering J., Mitchell E., Holder M.N., Guan X., Goddard M., Tegeler C., Beggs A.H., Childers M.K. (2012). Muscle function in a canine model of X-linked myotubular myopathy. Muscle Nerve.

[242] Buj-Bello A., Fougerousse F., Schwab Y., Messaddeq N., Spehner D., Pierson C.R., Durand M., Kretz C., Danos O., Douar A.M. (2008). AAV-mediated intramuscular delivery of myotubularin corrects the myotubular myopathy phenotype in targeted murine muscle and suggests a function in plasma membrane homeostasis. Hum. Mol. Genet..

[243] Elverman M., Goddard M.A., Mack D., Snyder J.M., Lawlor M.W., Meng H., Beggs A.H., Buj-Bello A., Poulard K., Marsh A.P. (2017). Long-term effects of systemic gene therapy in a canine model of myotubular myopathy. Muscle Nerve.

[244] Mack D.L., Poulard K., Goddard M.A., Latournerie V., Snyder J.M., Grange R.W., Elverman M.R., Denard J., Veron P., Buscara L. (2017). Systemic AAV8-Mediated Gene Therapy Drives Whole-Body Correction of Myotubular Myopathy in Dogs. Mol. Ther. J. Am. Soc. Gene Ther..

[245] Dupont J.B., Guo J., Renaud-Gabardos E., Poulard K., Latournerie V., Lawlor M.W., Grange R.W., Gray J.T., Buj-Bello A., Childers M.K. (2020). AAV-Mediated Gene Transfer Restores a Normal Muscle Transcriptome in a Canine Model of X-Linked Myotubular Myopathy. Mol. Ther. J. Am. Soc. Gene Ther..

[246] Phillips A., Belle A., Guo J., Ton J., Stinchcombe T., Buj-Bello A., Gray J.T. (2017). Nonhuman Primate Safety and Potency of an AAV Vector for XLMTM Produced by Transient Transfection at 500L. Mol. Ther..

[247] Kuntz N., Shieh P.B., Smith B., Bonnemann C.G., Dowling J.J., Lawlor M.W., Müller-Felber W., Noursalehi M., Rico S., Servais L. (2018). ASPIRO phase 1/2 gene therapy trial In X-linked myotubular myopathy: Preliminary safety and efficacy findings. Neuromuscul. Disord..

[248] Kuntz N., Shieh P.B., Smith B., Bonnemann C.G., Dowling J.J., Lawlor M.W., Müller-Felber W., Noursalehi M., Rico S., Servais L. (2018). ASPIRO phase 1/2 gene therapy trail in X-linked myotubular myopathy (XLMTM): Preliminary safety and efficacy findings. Mol. Ther..

[249] Kuntz N., Shie P.B., Smith B., Bonnemann C.G., Dowling J.J., Lawlor M.W., Mavilio F., Muller-Felber W., Noursalehi M., Rico S. (2018). Gene therapy for X-linked myotubular myopathy with AT132 (rAAV8-Des-hMTM1): Preliminary results from the ASPIRO phase-1/2 study. Hum. Gene Ther..

[250] Somanathan S., Calcedo R., Wilson J.M. (2020). Adenovirus-Antibody Complexes Contributed to Lethal Systemic Inflammation in a Gene Therapy Trial. Mol. Ther. J. Am. Soc. Gene Ther..

[251] Wilson J.M., Flotte T.R. (2020). Moving Forward After Two Deaths in a Gene Therapy Trial of Myotubular Myopathy. Hum. Gene Ther..

[252] Santiago-Ortiz J., Ojala D.S., Westesson O., Weinstein J.R., Wong S.Y., Steinsapir A., Kumar S., Holmes I., Schaffer D.V. (2015). AAV ancestral reconstruction library enables selection of broadly infectious viral variants. Gene Ther..

[253] Asokan A., Conway J.C., Phillips J.L., Li C., Hegge J., Sinnott R., Yadav S., DiPrimio N., Nam H.J., Agbandje-McKenna M. (2010). Reengineering a receptor footprint of adeno-associated virus enables selective and systemic gene transfer to muscle. Nat. Biotechnol..

[254] Yu C.Y., Yuan Z., Cao Z., Wang B., Qiao C., Li J., Xiao X. (2009). A muscle-targeting peptide displayed on AAV2 improves muscle tropism on systemic delivery. Gene Ther..

[255] Ying Y., Muller O.J., Goehringer C., Leuchs B., Trepel M., Katus H.A., Kleinschmidt J.A. (2010). Heart-targeted adeno-associated viral vectors selected by In Vivo biopanning of a random viral display peptide library. Gene Ther..

[256] Shen S., Horowitz E.D., Troupes A.N., Brown S.M., Pulicherla N., Samulski R.J., Agbandje-McKenna M., Asokan A. (2013). Engraftment of a galactose receptor footprint onto adeno-associated viral capsids improves transduction efficiency. J. Biol. Chem..

[257] Tarantal A.F., Lee C.C.I., Martinez M.L., Asokan A., Samulski R.J. (2017). Systemic and Persistent Muscle Gene Expression in Rhesus Monkeys with a Liver De-Targeted Adeno-Associated Virus Vector. Hum. Gene Ther..

[258] Pulicherla N., Shen S., Yadav S., Debbink K., Govindasamy L., Agbandje-McKenna M., Asokan A. (2011). Engineering liver-detargeted AAV9 vectors for cardiac and musculoskeletal gene transfer. Mol. Ther. J. Am. Soc. Gene Ther..

[259] Tulalamba W., Weinmann J., Pham Q.H., El Andari J., VandenDriessche T., Chuah M.K., Grimm D. (2020). Distinct transduction of muscle tissue in mice after systemic delivery of AAVpo1 vectors. Gene Ther..

[260] Choudhury S.R., Fitzpatrick Z., Harris A.F., Maitland S.A., Ferreira J.S., Zhang Y., Ma S., Sharma R.B., Gray-Edwards H.L., Johnson J.A. (2016). In Vivo Selection Yields AAV-B1 Capsid for Central Nervous System and Muscle Gene Therapy. Mol. Ther. J. Am. Soc. Gene Ther..

[261] Hakim C.H., Yue Y., Shin J.H., Williams R.R., Zhang K., Smith B.F., Duan D. (2014). Systemic gene transfer reveals distinctive muscle transduction profile of tyrosine mutant AAV-1, -6, and -9 in neonatal dogs. Mol. Ther. Methods Clin. Dev..

[262] Yang L., Li J., Xiao X. (2011). Directed evolution of adeno-associated virus (AAV) as vector for muscle gene therapy. Methods Mol. Biol..

[263] Yang L., Jiang J., Drouin L.M., Agbandje-McKenna M., Chen C., Qiao C., Pu D., Hu X., Wang D.Z., Li J. (2009). A myocardium tropic adeno-associated virus (AAV) evolved by DNA shuffling and In Vivo selection. Proc. Natl. Acad. Sci. USA.

[264] Qiao C., Zhang W., Yuan Z., Shin J.H., Li J., Jayandharan G.R., Zhong L., Srivastava A., Xiao X., Duan D. (2010). Adeno-associated virus serotype 6 capsid tyrosine-to-phenylalanine mutations improve gene transfer to skeletal muscle. Hum. Gene Ther..

[265] Li Y., Li J., Liu Y., Shi Z., Liu H., Wei Y., Yang L. (2019). Bat adeno-associated viruses as gene therapy vectors with the potential to evade human neutralizing antibodies. Gene Ther..

[266] Maheshri N., Koerber J.T., Kaspar B.K., Schaffer D.V. (2006). Directed evolution of adeno-associated virus yields enhanced gene delivery vectors. Nat. Biotechnol..

[267] Tse L.V., Klinc K.A., Madigan V.J., Castellanos Rivera R.M., Wells L.F., Havlik L.P., Smith J.K., Agbandje-McKenna M., Asokan A. (2017). Structure-guided evolution of antigenically distinct adeno-associated virus variants for immune evasion. Proc. Natl. Acad. Sci. USA.

[268] Li C., Wu S., Albright B., Hirsch M., Li W., Tseng Y.S., Agbandje-McKenna M., McPhee S., Asokan A., Samulski R.J. (2016). Development of Patient-specific AAV Vectors After Neutralizing Antibody Selection for Enhanced Muscle Gene Transfer. Mol. Ther. J. Am. Soc. Gene Ther..

[269] Sarcar S., Tulalamba W., Rincon M.Y., Tipanee J., Pham H.Q., Evens H., Boon D., Samara-Kuko E., Keyaerts M., Loperfido M. (2019). Next-generation muscle-directed gene therapy by in silico vector design. Nat. Commun..

[270] Piekarowicz K., Bertrand A.T., Azibani F., Beuvin M., Julien L., Machowska M., Bonne G., Rzepecki R. (2019). A Muscle Hybrid Promoter as a Novel Tool for Gene Therapy. Mol. Ther. Methods Clin. Dev..

[271] Porro F., Bortolussi G., Barzel A., De Caneva A., Iaconcig A., Vodret S., Zentilin L., Kay M.A., Muro A.F. (2017). Promoterless gene targeting without nucleases rescues lethality of a Crigler-Najjar syndrome mouse model. EMBO Mol. Med..

[272] Barzel A., Paulk N.K., Shi Y., Huang Y., Chu K., Zhang F., Valdmanis P.N., Spector L.P., Porteus M.H., Gaensler K.M. (2015). Promoterless gene targeting without nucleases ameliorates haemophilia B in mice. Nature.

[273] Powell S.K., Rivera-Soto R., Gray S.J. (2015). Viral expression cassette elements to enhance transgene target specificity and expression in gene therapy. Discov. Med..

[274] Bartel D.P. (2004). MicroRNAs: Genomics, biogenesis, mechanism, and function. Cell.

[275] Lee R.C., Feinbaum R.L., Ambros V. (1993). The *C. elegans* heterochronic gene lin-4 encodes small RNAs with antisense complementarity to lin-14. Cell.

[276] Kozomara A., Birgaoanu M., Griffiths-Jones S. (2019). miRBase: From microRNA sequences to function. Nucleic Acids Res..

[277] Lee E.J., Baek M., Gusev Y., Brackett D.J., Nuovo G.J., Schmittgen T.D. (2008). Systematic evaluation of microRNA processing patterns in tissues, cell lines, and tumors. RNA.

[278] Vechetti I.J., Wen Y., Chaillou T., Murach K.A., Alimov A.P., Figueiredo V.C., Dal-Pai-Silva M., McCarthy J.J. (2019). Life-long reduction in myomiR expression does not adversely affect skeletal muscle morphology. Sci. Rep..

[279] Brown B.D., Gentner B., Cantore A., Colleoni S., Amendola M., Zingale A., Baccarini A., Lazzari G., Galli C., Naldini L. (2007). Endogenous microRNA can be broadly exploited to regulate transgene expression according to tissue, lineage and differentiation state. Nat. Biotechnol..

[280] Roudaut C., Le Roy F., Suel L., Poupiot J., Charton K., Bartoli M., Richard I. (2013). Restriction of calpain3 expression to the skeletal muscle prevents cardiac toxicity and corrects pathology in a murine model of limb-girdle muscular dystrophy. Circulation.

[281] Geisler A., Jungmann A., Kurreck J., Poller W., Katus H.A., Vetter R., Fechner H., Muller O.J. (2011). microRNA122-regulated transgene expression increases specificity of cardiac gene transfer upon intravenous delivery of AAV9 vectors. Gene Ther..

[282] Qiao C., Yuan Z., Li J., He B., Zheng H., Mayer C., Li J., Xiao X. (2011). Liver-specific microRNA-122 target sequences incorporated in AAV vectors efficiently inhibits transgene expression in the liver. Gene Ther..

[283] Brown B.D., Venneri M.A., Zingale A., Sergi Sergi L., Naldini L. (2006). Endogenous microRNA regulation suppresses transgene expression in hematopoietic lineages and enables stable gene transfer. Nat. Med..

[284] Brown B.D., Cantore A., Annoni A., Sergi L.S., Lombardo A., Della Valle P., D’Angelo A., Naldini L. (2007). A microRNA-regulated lentiviral vector mediates stable correction of hemophilia B mice. Blood.

[285] Majowicz A., Maczuga P., Kwikkers K.L., van der Marel S., van Logtenstein R., Petry H., van Deventer S.J., Konstantinova P., Ferreira V. (2013). Mir-142-3p target sequences reduce transgene-directed immunogenicity following intramuscular adeno-associated virus 1 vector-mediated gene delivery. J. Gene Med..

[286] Carpentier M., Lorain S., Chappert P., Lalfer M., Hardet R., Urbain D., Peccate C., Adriouch S., Garcia L., Davoust J. (2015). Intrinsic Transgene Immunogenicity Gears CD8(+) T-cell Priming After rAAV-Mediated Muscle Gene Transfer. Mol. Ther. J. Am. Soc. Gene Ther..

[287] Boisgerault F., Gross D.A., Ferrand M., Poupiot J., Darocha S., Richard I., Galy A. (2013). Prolonged gene expression in muscle is achieved without active immune tolerance using microrRNA 142.3p-regulated rAAV gene transfer. Hum. Gene Ther..

[288] Belbellaa B., Reutenauer L., Messaddeq N., Monassier L., Puccio H. (2020). High levels of frataxin overexpression leads to mitochondrial and cardiac toxicity in mouse model. Mol. Ther. Methods Clin. Dev..

[289] Kraszewska I., Tomczyk M., Andrysiak K., Biniecka M., Geisler A., Fechner H., Zembala M., Stepniewski J., Dulak J., Jazwa-Kusior A. (2020). Variability in Cardiac miRNA-122 Level Determines Therapeutic Potential of miRNA-Regulated AAV Vectors. Mol. Ther. Methods Clin. Dev..

[290] Guo L., Zhang Q., Ma X., Wang J., Liang T. (2017). miRNA and mRNA expression analysis reveals potential sex-biased miRNA expression. Sci. Rep..

[291] Gao W., He H.W., Wang Z.M., Zhao H., Lian X.Q., Wang Y.S., Zhu J., Yan J.J., Zhang D.G., Yang Z.J. (2012). Plasma levels of lipometabolism-related miR-122 and miR-370 are increased in patients with hyperlipidemia and associated with coronary artery disease. Lipids Health Dis..

[292] Puzzo F., Colella P., Biferi M.G., Bali D., Paulk N.K., Vidal P., Collaud F., Simon-Sola M., Charles S., Hardet R. (2017). Rescue of Pompe disease in mice by AAV-mediated liver delivery of secretable acid alpha-glucosidase. Sci. Transl. Med..

[293] Paterna J.C., Moccetti T., Mura A., Feldon J., Bueler H. (2000). Influence of promoter and WHV post-transcriptional regulatory element on AAV-mediated transgene expression in the rat brain. Gene Ther..

[294] Brown B.D., Naldini L. (2009). Exploiting and antagonizing microRNA regulation for therapeutic and experimental applications. Nat. Rev. Genet..

[295] Dressman D., Araishi K., Imamura M., Sasaoka T., Liu L.A., Engvall E., Hoffman E.P. (2002). Delivery of alpha- and beta-sarcoglycan by recombinant adeno-associated virus: Efficient rescue of muscle, but differential toxicity. Hum. Gene Ther..

[296] Ayuso E., Blouin V., Lock M., McGorray S., Leon X., Alvira M.R., Auricchio A., Bucher S., Chtarto A., Clark K.R. (2014). Manufacturing and characterization of a recombinant adeno-associated virus type 8 reference standard material. Hum. Gene Ther..

[297] Prior H., Baldrick P., Beken S., Booler H., Bower N., Brooker P., Brown P., Burlinson B., Burns-Naas L.A., Casey W. (2020). Opportunities for use of one species for longer-term toxicology testing during drug development: A cross-industry evaluation. Regul. Toxicol. Pharmacol. RTP.

[298] EMA (2019). Guideline on Quality, Non-Clinical and Clinical Requirements for Investigational Advanced Therapy Medicinal Products in Clinical Trials. https://www.ema.europa.eu/en/documents/scientific-guideline/draft-guideline-quality-non-clinical-clinical-requirements-investigational-advanced-therapy_en.pdf.

[299] EMA (2011). ICH Guideline S6 (R1)–Preclinical Safety Evaluation of Biotechnology-Derived Pharmaceuticals. https://www.ema.europa.eu/en/documents/scientific-guideline/ich-s6r1-preclinical-safety-evaluation-biotechnology-derived-pharmaceuticals-step-5_en.pdf.

[300] Gao K., Li M., Zhong L., Su Q., Li J., Li S., He R., Zhang Y., Hendricks G., Wang J. (2014). Empty Virions In AAV8 Vector Preparations Reduce Transduction Efficiency And May Cause Total Viral Particle Dose-Limiting Side-Effects. Mol. Ther. Methods Clin. Dev..

[301] Deland F.H., North W.A. (1968). Relationship between liver size and body size. Radiology.

[302] Davidoff A.M., Ng C.Y., Zhou J., Spence Y., Nathwani A.C. (2003). Sex significantly influences transduction of murine liver by recombinant adeno-associated viral vectors through an androgen-dependent pathway. Blood.

[303] Ronzitti G., Gross D.A., Mingozzi F. (2020). Human Immune Responses to Adeno-Associated Virus (AAV) Vectors. Front. Immunol..

[304] Chicoine L.G., Montgomery C.L., Bremer W.G., Shontz K.M., Griffin D.A., Heller K.N., Lewis S., Malik V., Grose W.E., Shilling C.J. (2014). Plasmapheresis eliminates the negative impact of AAV antibodies on microdystrophin gene expression following vascular delivery. Mol. Ther. J. Am. Soc. Gene Ther..

[305] Mingozzi F., High K.A. (2017). Overcoming the Host Immune Response to Adeno-Associated Virus Gene Delivery Vectors: The Race between Clearance, Tolerance, Neutralization, and Escape. Annu. Rev. Virol..

[306] Meliani A., Boisgerault F., Hardet R., Marmier S., Collaud F., Ronzitti G., Leborgne C., Costa Verdera H., Simon Sola M., Charles S. (2018). Antigen-selective modulation of AAV immunogenicity with tolerogenic rapamycin nanoparticles enables successful vector re-administration. Nat. Commun..

[307] Mingozzi F., Anguela X.M., Pavani G., Chen Y., Davidson R.J., Hui D.J., Yazicioglu M., Elkouby L., Hinderer C.J., Faella A. (2013). Overcoming preexisting humoral immunity to AAV using capsid decoys. Sci. Transl. Med..

[308] Leborgne C., Barbon E., Alexander J.M., Hanby H., Delignat S., Cohen D.M., Collaud F., Muraleetharan S., Lupo D., Silverberg J. (2020). IgG-cleaving endopeptidase enables In Vivo gene therapy in the presence of anti-AAV neutralizing antibodies. Nat. Med..

[309] Jordan S.C., Lorant T., Choi J., Kjellman C., Winstedt L., Bengtsson M., Zhang X., Eich T., Toyoda M., Eriksson B.M. (2017). IgG Endopeptidase in Highly Sensitized Patients Undergoing Transplantation. N. Engl. J. Med..

[310] Elmore Z.C., Oh D.K., Simon K.E., Fanous M.M., Asokan A. (2020). Rescuing AAV gene transfer from neutralizing antibodies with an IgG-degrading enzyme. JCI Insight.

[311] Jonuschies J., Antoniou M., Waddington S., Boldrin L., Muntoni F., Thrasher A., Morgan J. (2014). The human desmin promoter drives robust gene expression for skeletal muscle stem cell-mediated gene therapy. Curr. Gene Ther..

[312] Lisowski L., Lau A., Wang Z., Zhang Y., Zhang F., Grompe M., Kay M.A. (2012). Ribosomal DNA integrating rAAV-rDNA vectors allow for stable transgene expression. Mol. Ther. J. Am. Soc. Gene Ther..

[313] Rosas L.E., Grieves J.L., Zaraspe K., La Perle K.M., Fu H., McCarty D.M. (2012). Patterns of scAAV vector insertion associated with oncogenic events in a mouse model for genotoxicity. Mol. Ther. J. Am. Soc. Gene Ther..

[314] GlobeNewswire (2017). Sarepta Therapeutics and Genethon Announce a Gene Therapy Research Collaboration for the Treatment of Duchenne Muscular Dystrophy. https://www.globenewswire.com/news-release/2017/06/21/1027114/0/en/Sarepta-Therapeutics-and-Genethon-Announce-a-Gene-Therapy-Research-Collaboration-for-the-Treatment-of-Duchenne-Muscular-Dystrophy.html.

